# Impact of Phase-Separated Janus-Type Formation on the Reversibility of Multicomponent Exsolved Nanoparticles from Complex Perovskites

**DOI:** 10.1021/acsnano.6c01371

**Published:** 2026-05-05

**Authors:** Blanca Delgado-Galicia, Andrés López-García, Catalina Elena Jiménez, Rosario Suarez-Anzorena, Marcus Bär, Virginia Pérez-Dieste, Ainara Aguadero, Jose A. Alonso, Inés Puente-Orench, Laura Almar, Alfonso J. Carrillo, José Manuel Serra

**Affiliations:** † Instituto de Tecnología Química (ITQ), Consejo Superior de Investigaciones Científicas-Universitat Politècnica de València, 46022 Valencia, Spain; ‡ Dept. Interface Design, Helmholtz-Zentrum Berlin für Materialien und Energie GmbH (HZB), Albert-Einstein-Str.15, 12489 Berlin, Germany; § Dept. X-ray Spectroscopy at Interfaces of Thin Films, Helmholtz-Institute Erlangen-Nürnberg for Renewable Energy (HI ERN), Albert-Einstein-Str. 15, 12489 Berlin, Germany; ∥ Department of Chemistry and Pharmacy, Friedrich-Alexander-Universität Erlangen- Nürnberg (FAU), Egerlandstr. 3, 91058 Erlangen, Germany; ⊥ ALBA Synchrotron, Carrer de la Llum 2-26, 08290 Barcelona, Spain; # 69570 Instituto de Ciencia de Materiales de Madrid, CSIC, Cantoblanco, Madrid 28049, Spain; ∇ Diffraction Group, Institut Laue-Langevin, 71 Ave des Martyrs, CS 20156, Grenoble cedex 9, France; ○ Instituto de Nanociencia y Materiales de Aragón (INMA-CSIC), C/Pedro Cerbuna, 12, 50009 Zaragoza, Spain

**Keywords:** exsolution, alloys, Janus nanoparticles, SOCs, reversibility

## Abstract

Solid oxide electrochemical cells (SOCs) benefit from exsolution-based electrocatalyst design, where nanoparticles anchored in perovskites enhance stability and activity. Two of the most transformative features of this technology are the ability to engineer multielemental alloy nanoparticles for tailored catalysis and the potential for *in situ* catalyst regeneration through redox-driven redissolution. However, the fundamental mechanisms governing these processes in complex, multicomponent systems remain poorly understood. In this work, the simultaneous exsolution of Fe, Ni, Co, and Cu from the fuel electrode material Sr_2_Fe_1.2_Co_0.1_Ni_0.1_Cu_0.1_Mo_0.5_O_6‑δ_ was investigated using *in situ* powder neutron diffraction and synchrotron-based near-ambient-pressure X-ray photoelectron spectroscopy (NAP-XPS), combined with advanced electron microscopy to capture morphological evolution. At 700 °C, Cu-rich nanoparticles dominate, consistent with Ellingham reducibility trends; however, higher temperatures favor the formation of Fe-enriched alloys, driven by the high availability of Fe cations. Conversely, prolonged reduction promotes the formation of phase-separated Janus-type nanoparticles, primarily due to Fe–Cu immiscibility. Interestingly, redox cycling tests revealed that nanoparticle composition dictates redissolution capacity. While homogeneous alloys exhibited total redissolution into the perovskite backbone and subsequent re-exsolution, Janus-type nanoparticles underwent irreversible transformation into pyramidal NiO nanoparticles via intermediate cubic mixed oxide structures during air exposure. These findings elucidate how temperature, time, and elemental composition govern exsolved nanoparticle chemistry, morphology, and regeneration, establishing design principles for inducing multimetal exsolution in complex oxides toward enhanced electrocatalytic performance in energy conversion technologies.

## Graphical abstract

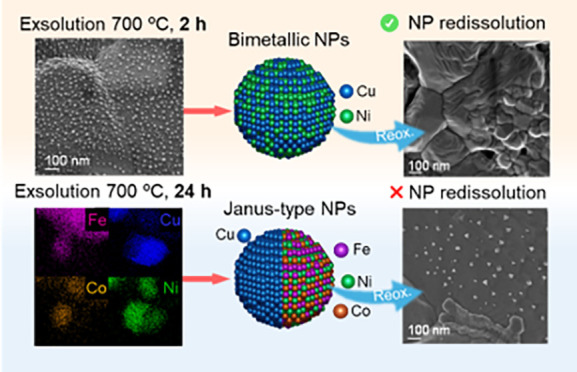

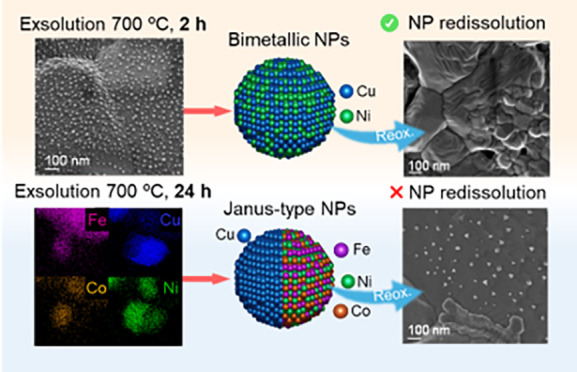

## 1 Introduction

Solid oxide fuel cells (SOFCs) and electrolyzers (SOECs) are pivotal technologies for clean energy conversion and storage, offering high efficiency and fuel flexibility.^1^ A key challenge in these devices lies in enhancing the performance and durability of the fuel electrode under harsh operating conditions (high temperature of operation, reducing atmospheres).^2^ In this context, exsolution, a process in which catalytically active nanoparticles emerge from a host lattice, has gained significant attention.^3–6^ It enables the *in situ* formation of stable, well-anchored nanoparticles that improve catalytic activity, redox stability, and resistance to coking and sulfur poisoning.^7,8^ As such, exsolution is arising as a powerful strategy to functionalize and optimize fuel electrodes for next-generation solid oxide devices.^9^ In the past decade, important advances have been demonstrated in the exsolution field, from the possibility of exsolving nanoparticles using electrochemical poling,^10^ plasma,^11^ or microwaves^12^ to the creation of nonmetallic nanoparticles^13,14^ or the exsolution of multicomponent metallic nanoparticles,^15^ which have unlocked unprecedented (electro)­catalytic functionalities with enhanced robustness. One of the key advantages of exsolution is the possibility of easily tuning the nanoparticle compositional and morphological properties by adjusting the processing conditions (external parameters)^16^ such as time, temperature,^17^ pressure,^18^ gas composition,^19^ or the intrinsic material properties such as porosity,^20^ strain,^21^ dislocations,^22^ crystal orientation,^23^ defects,^24^ A-site deficiency,^25,26^ doping,^27^ and B-site composition, among others.^26^ While most aspects affect particle size, shape, or dispersion, the latter significantly impacts the exsolution of bimetallic or multicomponent alloy nanoparticles, which exhibit improved catalytic activity over monometallic ones.^28^ However, multicomponent exsolution is not limited to metallic alloys, and other structures such as Janus-type^29,30^ or core–shell nanoparticles^31^ have been recently reported, extending its realm of application.

Bimetallic exsolution has attracted most of the attention,^32^ whereas the exsolution of multicomponent alloys has been scarcer due to its complexity. Probably the first work that dealt with the exsolution of three or more elements was by Santaya et al., who reported Ni–Co–Fe exsolution from a Sr_0.93_(Ti_0.3_Fe_0.56_Ni_0.07_Co_0.07_)­O_3−δ_ perovskite.^33^ Regarding the exsolution of more components, Shah et al. reported exsolution of five metals Ni, Fe, Co, Cu, and Pd from a LaFe_0.7_Ni_0.1_Co_0.1_Cu_0.05_Pd_0.05_O_3_ perovskite.^34^ Interestingly, and using thin-films of the same composition, Guo et al. obtained diverse complex structures via the so-called Exsolution Self Assembly (ESA) in Concentrated Complex Oxides (CCOs), ranging from Pd nanorods embedded in the oxide to Pd–Ni_*x*_Co_1–*x*_O core–shell nanoparticles.^35^ These works illustrated the wide range of possibilities for obtaining multicomponent alloyed exsolved nanoparticles. In terms of applications, multielemental NP exsolution has also proved to have remarkable effect in terms of improved thermocatalyic (e.g., dry reforming^36^) and electrochemical performance. Namely, for CO_2_ electrolysis in SOECs, both Fe–Co–Ni^26^ ternary and Fe–Co–Ni–Cu quaternary^37^ exsolutions have demonstrated enhanced electrocatalytic activity confirmed by a decreased polarization resistance. In SOFC mode, very recently, Lach et al. demonstrated the positive effects of Fe–Ni–Co exsolution from Sm_0.9_Ba_0.9_Mn_1.8–x_Fe_*x*_Co_0.1_Ni_0.1_O_5+δ_ nanofiber perovskites with polarization resistance of 0.046 Ω cm^2^ at 800 °C.^38^ These recent works demonstrate the positive impact of multielemental alloy exsolutions in electrochemical devices. However, several aspects of this complex phenomenon remain unclear, particularly the diffusion of several cations from the oxide lattice toward the surface, where they nucleate as nanoparticles. In particular, most of them are related to the different degrees of reducibility of these cations, which can strongly affect the composition of the multielement nanoparticles. In addition, the cationic diffusivities of each element can also affect nucleation and vary with respect to the host perovskite oxide. There is still no clear consensus on how the exsolution mechanism takes place. Kwon et al. demonstrated through experiments and Density Functional Theory (DFT) calculations that the favorable thermodynamic pathway is the separate exsolution of Co and Ni nanoparticles, which later merge on the surface to form bimetallic alloys.^39^ Regarding more fundamental aspects of exsolution, Bonkowski et al., supported by DFT calculations, proposed that the reduction of the exsolvable cations (Ni^2+^ in their study) occurs within the oxide and that Ni^0^ metal species are the ones that migrate to the surface to generate the nanoparticles.^40^ In the context of multicomponent exsolution, a fundamental mechanism has yet to be proposed; however, experimental results have identified specific trends linking cation reducibility to elemental composition.

In this context, the perovskite family Sr_2_Fe_1.5_Mo_0.5_O_6‑δ_ has proved to be a versatile oxide for multicomponent exsolution. This perovskite exhibits remarkable properties as a fuel electrode in SOC devices^41,42^ and has served as a valuable platform for exploring alloy exsolution.^43,44^ The focus has been on demonstrating how to tune the properties of ternary alloyed (Fe–Ni–Co) nanoparticles, with particular attention to understanding the effects of external treatments on their composition. It has been established that Ni tends to be the predominant species and that increasing temperature facilitates the exsolution of higher amounts of Fe.^26^ *In situ* synchrotron characterization proved this trend and the reversibility of the Ni–Co–Fe nanoparticles. Interestingly, a second exsolution treatment after redissolution leads to nanoparticles enriched in Fe.^45^ Finally, we also proved the effect of high pressure in ternary alloyed exsolution, which led to the exsolution of Fe-rich nanoparticles at pressures around 50 bar.^18^ These works demonstrated the high degree of compositional variability in the ternary alloyed nanoparticles and provide processing guidelines that enable Fe enrichment. In particular, high temperatures, medium pressures, or the application of redox cycles facilitated the exsolution of Fe, a cation less thermodynamic favorable to exsolve than Co, and especially, Ni. Building on our previous results, we now explore the exsolution of nanoparticles with four components, in which Cu, a cation with higher reducibility than Ni, is incorporated into the lattice, which might alter the previously reported trends. The objective is to assess how external processing parameters alter the composition of these quaternary multielemental exsolved nanoparticles. For that reason, the effects of time, temperature, and redox cycling have been studied in perovskites with the formula Sr_2_Fe_1.2_Co_0.1_Ni_0.1_Cu_0.1_Mo_0.5_O_6‑δ_. We utilized a set of techniques combining high-resolution electron microscopy, synchrotron-based *in situ* near-ambient-pressure X-ray photoelectron spectroscopy (NAP-XPS), and neutron diffraction to prove the composition of these nanoparticles and their reversibility, and the impact that external parameters have on the composition, size, shape, and dispersion of these promising nanocatalysts. These materials were further tested using electrochemical impedance spectroscopy to demonstrate the enhanced electrocatalytic properties of exsolution-functionalized electrodes in the context of SOC devices.

## 2 Results and Discussion

### 2.1 Sr_2_Fe_1.2_Co_0.1_Ni_0.1_Cu_0.1_Mo_0.5_O_6‑δ_ Physicochemical Properties

The Sr_2_Fe_1.2_Co_0.1_Ni_0.1_Cu_0.1_Mo_0.5_O_6‑δ_ crystal structure was first analyzed via *in situ* neutron powder diffraction (NPD) analyses, exemplified here at the pristine state (Figure 1a) and after exposure to vacuum at 700 °C (Figure 1b). The pristine sample crystallized in a cubic structure with *Fm*3̅*m* space group, without the presence of impurities or secondary phases. The heating treatment in vacuum caused the reduction of the material, as evidenced by the shift of the reflections to lower 2θ values, indicating an expansion of the lattice—obtained from Rietveld refinements—from 470.3 to 479.3 Å^3^ (Table 1). The Rietveld refinements were also employed to determine the initial oxygen vacancies (V_O_^••^) in the pristine material (oxygen occupancy, *O*_occ_ = 5.87; δ = 0.13) that significantly increased after reduction treatment (*O*_occ_ = 5.26; δ = 0.74) due to the formation of intrinsic V_O_^••^. Notably, several additional reflections appeared after reduction treatment corresponding to the Bragg positions of the Sr_2_Fe_1.5_Mo_0.5_O_6‑δ_ double-perovskite structure, whereas they were absent in the pristine material. This behavior suggests the emergence of superstructure reflections and, therefore, the development of long-range order characteristic of a double-perovskite arrangement upon reduction, implying that the pristine state might exhibit only limited long-range or short-range order and thus may not correspond to a true double perovskite. Consistent with these results, the structural parameter, *x*_*(*O*)*_, decreased from 0.251 to 0.239, reflecting a shift in the anion sublattice, associated with the reduction of the B-site cations and the consequent increase in their ionic radii, which triggers the lattice expansion (Table 1).

**Figure 1:**
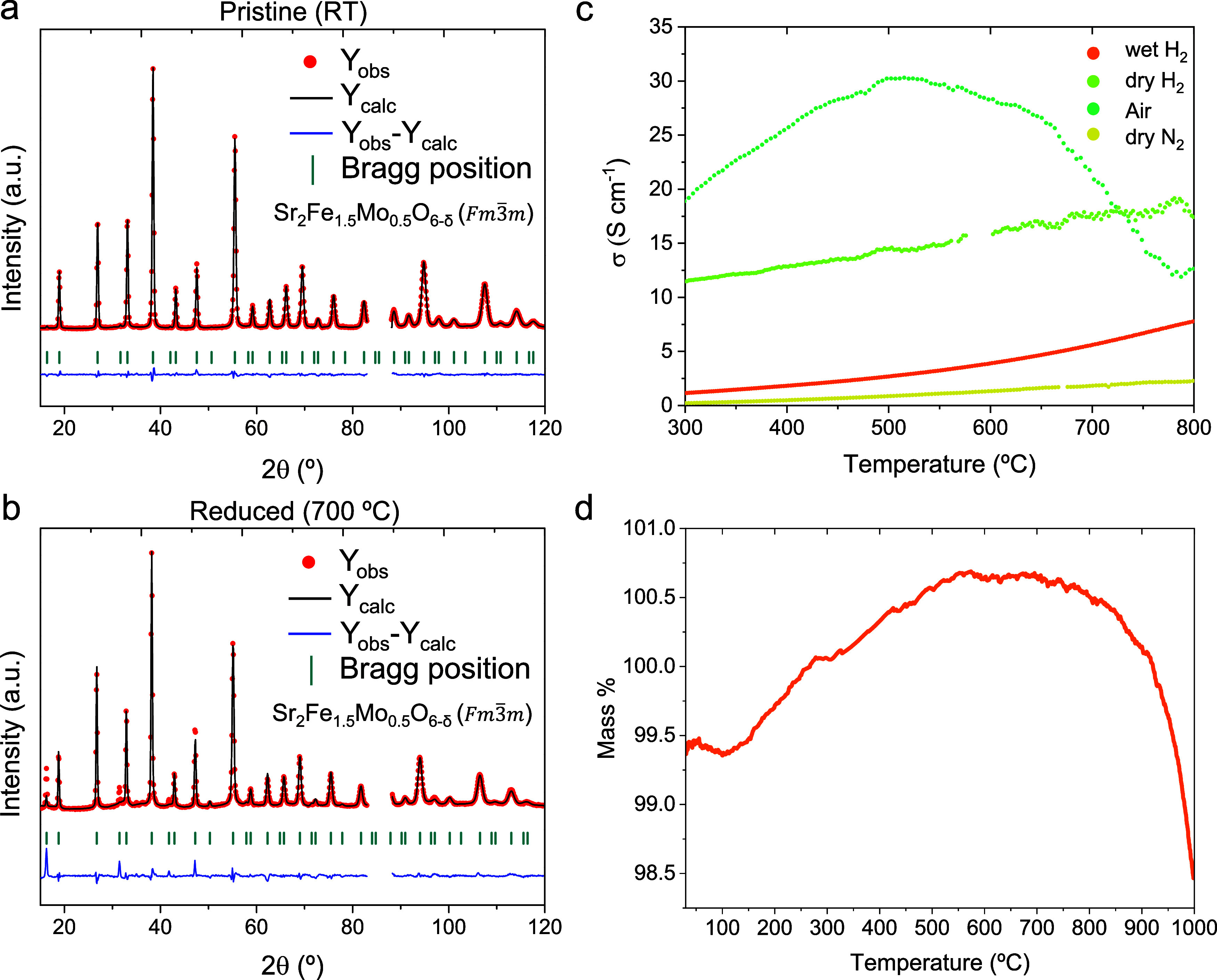
Rietveld refinements of neutron diffraction for (a) pristine and (b) 700 °C reduced in vacuum conditions of Sr_2_Fe_1.2_Co_0.1_Ni_0.1_Cu_0.1_Mo_0.5_O_6‑δ_ based on cubic Sr_2_Fe_1.5_Mo_0.5_O_6‑δ_ structure (*Fm*3̅*m*). The region between 2θ = 83–88° was excluded due to a detector set failure. (c) Electrical conductivity measurements under different atmospheres, namely, air, dry H_2_, wet H_2_, and dry N_2_. (d) Thermogravimetric analysis depicting the mass change (%) as a function of temperature under constant air flow (50 mL min^– 1^).

**Table 1:** Crystallographic Parameters for Pristine Sr_2_Fe_1.2_Co_0.1_Ni_0.1_Cu_0.1_Mo_0.5_O_6‑δ_ and after Reduction at 700 °C in Vacuum Conditions, Obtained through Rietveld Refinement of the Neutron Diffraction Results.^a^

sample label	lattice parameter, *a* (Å)	cell volume, *V*_cell_ (Å^3^)	oxygen occupancy, *O*_occ_	structural parameter, *x*_(O)_
Pristine	7.7764(3)	470.26(3)	5.87(2)	0.251(2)
Red.700 °C	7.825(1)	479.2(1)	5.26(2)	0.239(2)

a In the space group *Fm*3̅*m* (#225), Sr atoms are located at 8c (1/4,1/4,1/4) positions; Fe1 is at 4a (0,0,0), (Fe2,Ni,Cu,Co,Mo) are distributed at random at 4b (1/2,1/2,1/2) and O at 24e (x,0,0) Wyckoff sites.

Conductivity measurements under different atmospheres (wet H_2_, dry H_2_, air, and dry N_2_) are depicted in Figure 1c. Under air conditions, the material exhibits a typical conductivity for this family of perovskites,^26^ which is attributed to a small polaron hopping conduction mechanism. To understand the behavior, it is worthwhile to consider the thermogravimetric results first (Figure 1d). Conductivity increases up to a point (∼650 °C), where the mass percentage represented in TG also increases. This weight gain (∼1.3%) is ascribed to the reoxidation of the perovskite. As obtained from NPD, the pristine sample shows an initial oxygen deficiency; therefore, these initial oxygen vacancies are then refilled under these conditions, with the concomitant oxidation of B-site cations as the temperature increases. During this phase, conductivity rises with increasing temperature because of the thermal activation involved in small polaron hopping; at higher temperatures, polarons move more freely between charge carriers. When temperatures exceed 700 °C, the mass gain decreases markedly by 2.22%, as structural oxygen is lost, creating oxygen vacancies. At the same time, metals are reduced to lower oxidation states as temperature increases. Consequently, the number of charge carriers (holes in p-type conductivity) and polaron hopping are no longer the primary mechanisms for conductivity. For dry and wet H_2_ and N_2_ atmospheres, the conductivity aligns with that of a semiconductor, increasing with temperature as expected. Notably, SFCNCuMo shows higher conductivity in H_2_ than in N_2_. This perovskite can act as a redox electrode, functioning as a fuel or air electrode, evidenced by its high conductivity in air (20.9 S/cm at 700 °C) and dry H_2_ (17.25 S/cm at 700 °C).

### 2.2 Processing Effects on Multicomponent Nanoparticle Exsolution

#### 2.2.1 Temperature Dependence

The pristine material was subjected to a reductive treatment at different temperatures, ranging from 600 to 900 °C, to drive nanoparticle exsolution. Figure S1a shows the XRD results after each temperature treatment. Upon heating in a reductive (5% H_2_) atmosphere, the XRD reflections shifted progressively to lower 2θ values, indicative of lattice expansion. The unit-cell volume of the main phase was determined via Rietveld refinement of the cubic perovskite (*Fm*3̅*m*) as aforementioned (PDF #01-072-6388), Table S1, showing a progressive increase up to 800 °C, due to the reduction of B-site metal transition cations and the consequent increase in their ionic radii. At 800 °C, a Ruddlesden–Popper (RP) phase (Sr_3_FeMoO_7_) formed, with a peak at 31.6°, becoming the main phase at 900 °C. This crystallographic transition has been previously observed for similar compositions.^18,26,45^ Interestingly, metallic peaks at 44.6° corresponding to exsolution were only inferred at 900 °C, which could be ascribed to a larger size of the exsolved nanoparticles that allowed their detection. It is noted that the unit-cell volume determined by XRD was ca. 2% higher than that measured by NPD. This discrepancy can be caused by multiple factors (instrument calibration, different sample batches, preferred orientations, and refinement methods), but more likely due to the different treatments employed for reduction. NPD diffraction analyzed the sample reduced under vacuum and XRD studied 5% H_2_-reduced materials, which might imply higher reduced state. However, because all exsolved samples were measured under identical conditions and with the same equipment, the overall expansion trends remain consistent.

HRFESEM confirmed the presence of exsolved nanoparticles after each reduction treatment. Figure S1b shows the morphological differences between exsolved nanoparticles. In the range of 700 to 900 °C, the size of the nanoparticles increases with temperature from 15.6 to 36.5 nm (see Figure S2), while the population decreases from 745 to 109 nanoparticles/μm^2^ (data summarized in Figure S2e). This behavior is generally characteristic of exsolution as increasing the temperature results in larger nanoparticles and lower dispersions.^8,26^ At 600 °C, the average particle size is quite similar to that at 700 °C considering the deviation (18.0 vs 15.6 nm), while the population is notably lower (148 and 745 nanoparticles/μm^2^). This can be explained by the fact that deeper cations require more energy to diffuse and migrate to the surface to form nanoparticles than the superficial ones, which is attributed to the higher temperature of reduction treatment. These results support the following tests in this study, specifically those related to time-dependent exsolution and electrochemical characterization which were conducted mainly at 700 °C.

Next, exsolution conditions were set to 6 h and two temperatures 700 and 900 °C, to assess the phase composition evolution of the exsolved nanoparticles. Figure S3 shows the diffractograms at both temperatures. At 700 °C, the material reduces, retaining its cubic structure without evident crystallographic transitions. At 900 °C, a partial crystallographic transition occurs, forming cubic, metallic, and molybdates phases. HRTEM analyses (Figure 2a,b) reveal the exsolution characteristic nanoparticle anchoring to the host oxide in both cases. Figure S3b,c depicts HRFESEM micrographs and the size distribution for both samples, indicating that nanoparticle size increases with temperature, as previously shown, with mean sizes of 21 nm for 700 °C/6 h, and 32 nm for 900 °C/6 h. In addition, temperature not only affected nanoparticle size but also the composition, as summarized in Figure 2c. This figure displays the elemental composition of the nanoparticles obtained from the XEDS analyses shown in Figure 2d,e. These analyses indicated that both treatments could lead to the formation of exsolved nanoparticles composed of Cu–Ni–Co–Fe, as also recently reported by Liu et al.,^46^ but with remarkable compositional differences. Importantly, with the resolution of the XEDS mappings obtained here, it was possible to discern the formation of a quaternary alloys at 900 °C, but other specific treatments led to the formation of phase-separated (Janus-type) nanoparticles, as will be shown in the following results. The reductive treatment at 700 °C/6 h generates exsolved nanoparticles rich in Cu (57%), followed by Ni (33%) and scarcer amounts of Fe (7%) and Co (3%). On the other hand, by increasing the temperature up to 900 °C, the composition shifted depicting a remarkable Fe enrichment (58.5%), followed by Ni (18.5%), Co (17%), and Cu (6%). Similar trends were recently reported, where higher temperatures led to higher Fe content in the nanoparticles; however, Fe enrichment occurred to a lesser extent.^26^ This altered trend in the elemental composition with temperature might be explained by a change in the rate controlling the regime for exsolution with temperature. The exsolution compositional trend at 700 °C, following Cu > Ni > Fe > Co, mainly reflects the thermodynamic control of reducible B-site cations. This is illustrated in Figure S4, which displays the Ellingham plot for the reduction of CuO, NiO, FeO, and CoO by H_2_ into their metallic forms. As shown, Cu has the most negative Δ*G* (≈ −140 to −150 kJ/mol), indicating that CuO is the easiest to reduce with H_2_. The plot suggests a reducibility order of Cu > Ni > Co > Fe, with FeO reduction even showing positive Δ*G* values within the analyzed temperature range. Thus, thermodynamics indicates that Cu and Ni are more reducible, aligning with their higher concentration in the exsolved nanoparticle. Fe needs higher temperatures for reduction, but Cu remains the most reducible even at temperatures higher than 1000 °C. Hence, the higher Fe concentration at 900 °C can be explained through two synergistic factors. First, higher temperatures favor the reduction of Fe cations into Fe metallic species. Second, Fe is present in the perovskite in 4-fold higher amount with respect to the sum of the three other exsolvable cations (Co, Ni, and Cu). Thus, under the adequate thermodynamic conditions (*i.e*. increased temperatures), the higher availability of Fe cations dominate the multimetallic exsolution. It is worth noting that, at this temperature, a significant fraction of the material transforms into an RP phase (∼83.6%), likely Sr_3_FeMoO_7_, which introduces a Fe-rich structural environment that can further promote Fe exsolution. In this Fe-rich environment, it seems more plausible, and according to the XEDS in Figure 2e, that minor contribution of Co, Ni, and Cu led to the formation of an alloy. However, in situations in which Cu is predominant in the NP, increasing amounts of Fe, Co, and Ni led to phase-separated Janus-type nanoparticles. This behavior is explained in the context of metal miscibility in alloys. Specifically, Fe–Cu alloys exhibit limited solubility in intermediate compositions (equal proportion of metals), generally leading to phase-separated (FCC + BCC) metallic phases.^47,48^

**Figure 2:**
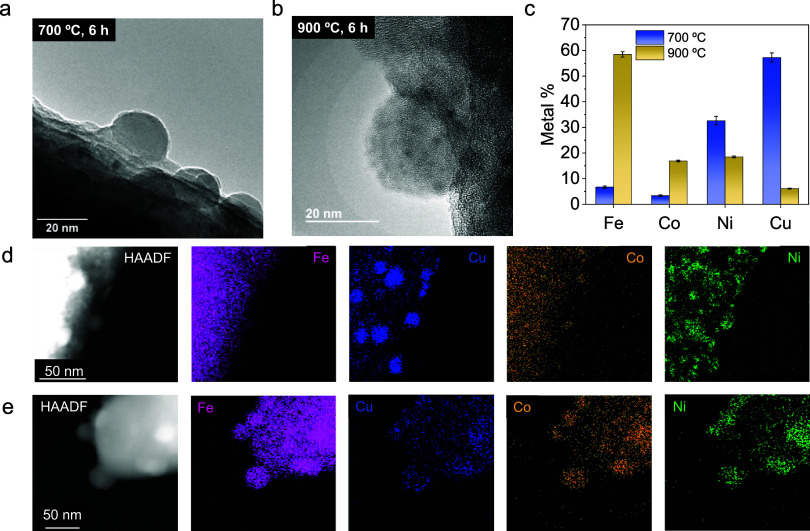
HRTEM images for Sr_2_Fe_1.2_Co_0.1_Ni_0.1_Cu_0.1_Mo_0.5_O_6−δ_ after exsolution at (a) 700 and (b) 900 °C for 6 h in 5% H_2_ in Ar. (c) Metal content in the nanoparticles after 700 and 900 °C exsolution treatments. HAADF-STEM and XEDS mapping analysis for exsolvable metals for (d) 700 °C/6 h and (e) 900 °C/6 h.

Higher temperatures also accelerate exsolution and cation diffusion, leading to larger nanoparticle sizes. However, the current data do not allow a complete explanation of the multielemental nanoparticle exsolution mechanism. Notably, the trends observed at 700 °C emphasize the importance of thermodynamics of the B-site cations, cation availability, and the significant influence of temperature on the composition of multimetallic exsolved nanoparticles. For example, Lui et al. reported quaternary alloyed nanoparticles with a composition where Co (∼35%) and Ni (∼30%) exceeded Cu (∼15.5%) and Fe (∼13.5%), formed during exsolution at 800 °C for 2 h in 5% H_2_/N_2_. This appears to be an intermediate stage before the results described here.^46^

#### 2.2.2 Time Dependence

The material was also subjected to different time treatments (2, 6, and 24 h), all performed at 700 °C in 5% H_2_ in Ar. XRD data in Figure S5a show the enhanced reduction of the material when increasing treatment time, as evidenced by the 2θ shifts to lower values due to lattice expansion. Contrarily to temperature effect, longer reduction times did not cause RP phase formation. Influence of time on the nanoparticle exsolution is evidenced by the HRFESEM micrographs and image analyses (Figure S5b). Longer exposure times significantly affected the dispersion, which decreased from ca. 750 nanoparticles/μm^2^ at 2 h to ca. 400 nanoparticles/μm^2^ after 24 h. Furthermore, the size distribution is remarkably wider, reaching a maximum frequency at ca. 12–15 nm, but showing relevant frequencies up to 25 nm (Figure S6).

The influence of time on the nanoparticle composition was also considered. HRTEM analyses (Figure 3a) reveals the anchoring of exsolved nanoparticles into the lattice after different exposure times. Figure 3b shows the compositional changes and the percentage of each metal in the exsolved nanoparticles. All four metals were exsolved after 2 h although the evolution and composition of the nanoparticles can be seen and better characterized after longer exposure times. For the 24 h test, the exsolution results in phase-separated Janus-type nanoparticles, where all four metals are present. This Janus particle is characterized by two compositionally different regions: one region mainly composed of Cu and a second region by the other three elements (Fe, Co, and Ni), generating specific-sided catalysts. Thus, at 700 °C, Cu does not alloy with the other three metallic components, consistent with other reported works.^29^ Although the XEDS results after 24 h (Figure 3e) are more prominent due to the increased detectable metal amount, the possibility of Janus nanoparticles being formed from the beginning of exsolution cannot be ruled out, as data from 6 h already showed the separation of the two different regions. In all three study durations, Cu remains the element with the highest concentration in the nanoparticles, ca. ≥ 50%, consistent with the thermodynamic considerations. Notably, the 24-h treatment led to a 3-fold increase in Fe concentration in the nanoparticles, primarily at the expense of Ni. These results demonstrate, for the first time, the possibility of generating Janus-type multimetallic nanoparticles via exsolution. In this specific case, the favorable temperatures for Cu exsolution caused one of the phases to be mostly formed by this element, whereas the other half of the spherical nanoparticle is composed of a ternary alloy of Fe–Co–Ni.

**Figure 3:**
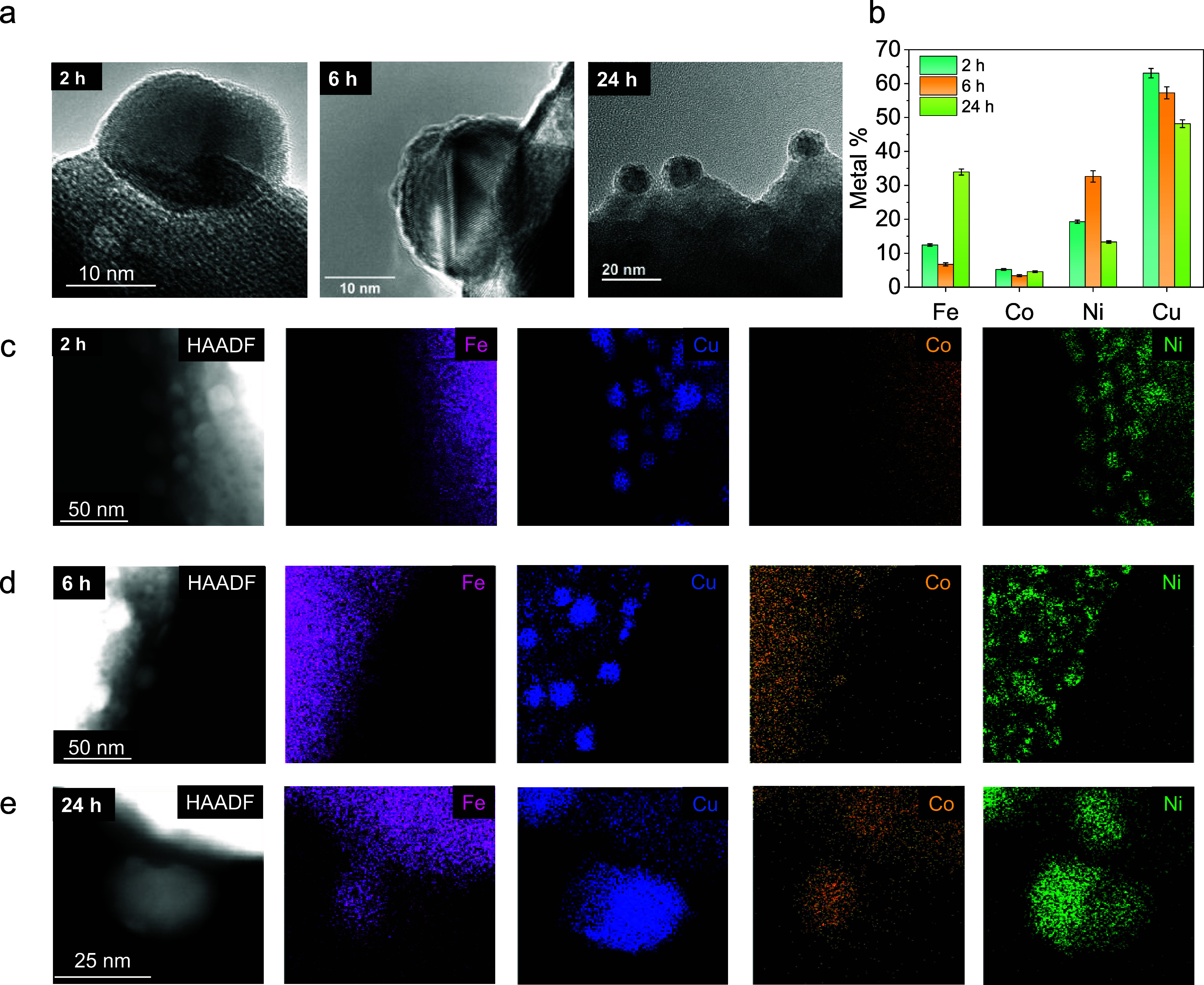
Data for exsolution at 700 °C for 2, 6, and 24 h in 5% H_2_ in Ar. (a) HRTEM images for the three exsolutions. (b) Bar diagram of metal content in the nanoparticles. HAADF-STEM and XEDS mapping analyses for exsolvable metals for (c) 700 °C/2 h, (d) 700 °C/6 h, and (e) 700 °C/24 h.

### 2.3 Exsolution Surface Property via *In Situ* NAP-XPS

Next, synchrotron-based *in situ* NAP-XPS was utilized to confirm the multimetallic nanoparticle formation under reducing conditions. For these sets of experiments, button cells (Figure S7) with a similar configuration as those employed for the electrochemical testing (see Section 2.5) were employed. Namely, the material was screen-printed as a composite fuel electrode together with CGO (60:40). The NAP-XPS experiment consisted in three main steps. First, a cleaning step at 600 °C under a pure O_2_ atmosphere to remove adventitious carbon species (see Figure S8). After this step, the core levels of Sr 3d, O 1s, Mo 3d, Cu 2p, Co 2p, Ni 2p, and Fe 2p were recorded (Figure 4a–g), which denotes the pristine stage (Prist), *viz*, the oxidized state of the material before exsolution. After this step, the cell was cooled down to 300 °C, followed by a change to H_2_ atmosphere, until reaching the target temperature (600 °C). From this point, the cell was heated under reducing conditions following the changes in the valence band signal, Figure 4h. Here, the objective was to identify the temperature at which metallic phases emerge by monitoring the appearance of the Fermi Edge (E_F_). The density of states at the E_F_ started to emerge between 450 and 475 °C, and it was clearly identified at 510 °C (Figure 4h), denoting the presence of metals at the surface. After this point, the core levels in the reduced (Red.) state were obtained. The formation of metallic phases was corroborated for the four exsolvable components, that is, Cu 2p (Figure 4a), Ni 2p (Figure 4b), Co 2p (Figure 4c), and Fe 2p (Figure 4d). For Cu and Ni the extent of reduction was higher than for Co, and especially, for Fe, as denoted by the relative areas of the metallic and oxidized species. It is important to note that, under reducing conditions, the spectra shifted approximately 1 eV to higher binding energy values, as indicated by the dashed lines in Figure 6f. This change is attributed to a Fermi-level shift within the perovskite band gap when transitioning from oxidizing to reductive atmospheres, as previously reported.^45,49^ These results confirmed the exsolution of the four B-site cations and corroborated the exsolution tendencies in the first reduction step. Namely, Cu and Ni have a higher level of reducibility, which facilitates the formation of exsolvable nanoparticles compared to Co and especially, to Fe. NAP-XPS also provided useful information about the other components present on the oxide surface. The reduction treatment also induced changes in the Mo, which partially reduced from Mo^6+^ to Mo^5+^ (Figure 4f). This is an indication of partial transformation of the double perovskite to an RP phase at the surface, as previously described.^45^ The deconvolution of the Mo species is shown in Figure S9. From these data, we could extract that 34.6% of the Mo^6+^ was reduced to Mo^5+^. Figure S10 shows the deconvolution of O 1s spectra in the pristine and reduced states. The O 1s spectra were fitted with three main components^50^: one at 532 eV attributed to adsorbed oxygen species at the surface, a peak at around 530 eV ascribed to oxygen in the outer-surface layer of the perovskite structure layer, and a third one being around 528 eV which is related to lattice oxygen. The deconvolution of O 1s spectrum shows a decrease in the intensity of the oxygen lattice component after reduction. This is an indication of the formation of oxygen vacancies upon reduction in H_2_, a key step prior to nanoparticle exsolution.^21^ In this step, lattice oxygen reacts with H_2_ forming water and generating oxygen vacancies (V_O_^••^), eq 1 in Kröger–Vink notation

1 $$H_{2}\left(g\right)+O_{O}^{x}\to H_{2}O\left(g\right)+V_{O}^{••}+2e^{\prime }$$

Sr 3d was fitted (Figure S11) using two spin–orbit doublet components: one at lower binding energies (Sr 3d_5/2_ at ∼132.2 eV) related to Sr in the perovskite lattice and the doublet at Sr 3d_5/2_ ∼ 133.5 eV, ascribed to surface species.^51^ This enables calculating the Sr “surface/bulk” ratio, which was 1.4 and 2.0 for the pristine and reduced samples, respectively. These values denoted a clear Sr enrichment in both states, which even increased upon reduction. This effect is commonly reported in Sr-rich perovskites.^52^ Please note that a broader peak shape was assumed for the surface component to accommodate the larger structural disorder (due to variations in bond angles and bond lengths) at the surface.^49^ Namely, for the reduced Mo^5+^ species (Figure S9), adsorbed and surface oxygen species (Figure S10) and surface Sr species (Figure S11) a less ordered structure with a variety of different bond angles and/or distances compared to that of the Mo^6+^ species, lattice oxygen species and lattice Sr species, respectively, is expected, which justifies the somewhat broader peak shape of the respective fit component.

**Figure 4:**
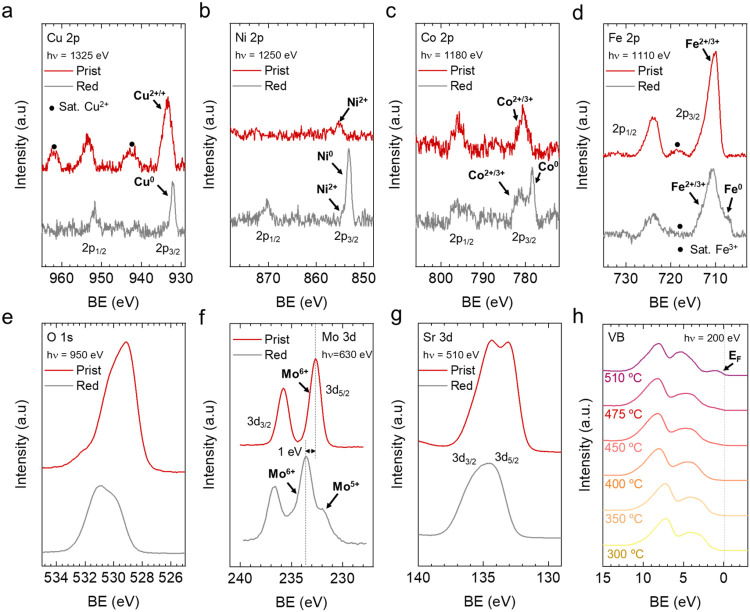
NAP-XPS spectra of (a) Cu 2p, (b) Ni 2p, (c) Co 2p, (d) Fe 2p, (e) O 1s, (f) Mo 3d, and (g) Sr 3d acquired *in situ* at 600 °C in pristine (“Prist”, 0.2 mbar O_2_), and ∼510 °C reduced (“Red”, 0.1 mbar H_2_) conditions. The used photon energies (to result in a similar electron kinetic energy of ∼400 eV) are indicated in the panels. A constant offset is added to the spectra for improved clarity. (h) Valence band (VB) spectra under heating conditions in 0.1 mbar H_2_ atmosphere. For (a–d), the background was subtracted to linearize the baseline.

### 2.4 Insights on Multicomponent Exsolution Reversibility

Potential catalyst regeneration through exsolution reversibility is one of the main features of this nanoparticle fabrication route.^53^ To evaluate this aspect, two different oxidation treatments for nanoparticle redissolution processes were tested, varying the redox exposure time. Figure 5 depicts the information regarding the first case, where a sample exsolved at 700 °C for 2 h is afterward subjected to a reoxidation process during 24 h in air at 800 °C. This higher temperature value (800 °C) was selected for enhancing the reoxidation kinetics. Figure 5b shows a complete redissolution of the exsolved nanoparticles back to the host oxide bulk. It is worth noting the remaining holes on the surface, suggesting a rearrangement of the surface around these nanoparticles during reoxidation, as previously reported in similar materials.^45^ *In situ* NAP-XPS was also utilized to analyze the surface property after a reoxidation treatment performed directly after exsolution, see Figure S12. There, it can be seen that after exposure to pure (0.2 mbar) O_2_, the metallic components of Ni 2p, Cu 2p, Co 2p, and Fe 2p vanished, ascribed to the redissolution of metallic Co, Fe and Ni, and Cu species into the bulk lattice as a cation. To further corroborate the total redissolution, Figure S13a shows a magnified view of the XRD pattern is shown, with the (111) and (200) NiO main reflections (37.3 ° and 43.3 °) indicated by dashed lines, confirming the absence of any detectable NiO phase. In addition, high-magnification HRFESEM imaging (Figure S13b) and HAADF-XDS (Figure S13c) analyses of the oxidized surface further support the complete redissolution of the exsolved nanoparticles, with no evidence of NiO particles present on the surface. After the complete redissolution process, the samples were further subjected to a reduction treatment under 5% H_2_, 700 °C for 2 h. The HRFESEM micrographs in Figure 5c confirmed the successful re-exsolution, thus the regeneration of exsolved nanoparticles after full redissolution. These micrographs’ statistical analyses (Figure S14) revealed that the nanoparticle size diminished from 15.6 to 9.2 nm in the second re-exsolution. Additionally, the re-exsolution process helped in increasing the nanoparticle population, namely, from 745 to 1131 nanoparticles/μm^2^ after re-exsolution. This effect was already reported by Lv et al. with RuFe NPs exsolved from Sr_2_Fe_1.4_Ru_0.1_Mo_0.5_O_6‑δ_, which, after four redox cycles, increased in population by 3.6 times caused by surface Ru enrichment.^54^ Interestingly, the opposite behavior was reported with Sr_2_FeNi_0.2_Co_0.2_Mn_0.1_Mo_0.5_O_6‑δ_ in which one redox cycle resulted in a decrease in the population. In that specific case, and noting that the oxidation step was of 24 h, the redox treatment caused Fe enrichment.^45^ From these works is evident that the nature and reducibility behavior of the cations involved in the exsolution have a significant impact over the reversibility process. In order to discern possible alterations in the composition of the re-exsolved nanoparticles, Figure 5d,e compares the elemental distribution of the exsolved and re-exsolved nanoparticles. The XEDS maps revealed that the re-exsolved nanoparticles are mainly formed by Cu, in line with the Ellingham reducibility trends, while exsolved (700 °C/2 h) nanoparticles show significant amounts of both Cu and Ni. Figure S15 depicts the XRD diffractograms for the three states (exsolved, oxidized, re-exsolved), confirming that the cubic phase is retained, without phase alterations or secondary phases emergence. The zoom-out image provides the lattice contraction upon oxidation and the subsequent lattice expansion after the second reductive treatment, confirming the redox process.

**Figure 5:**
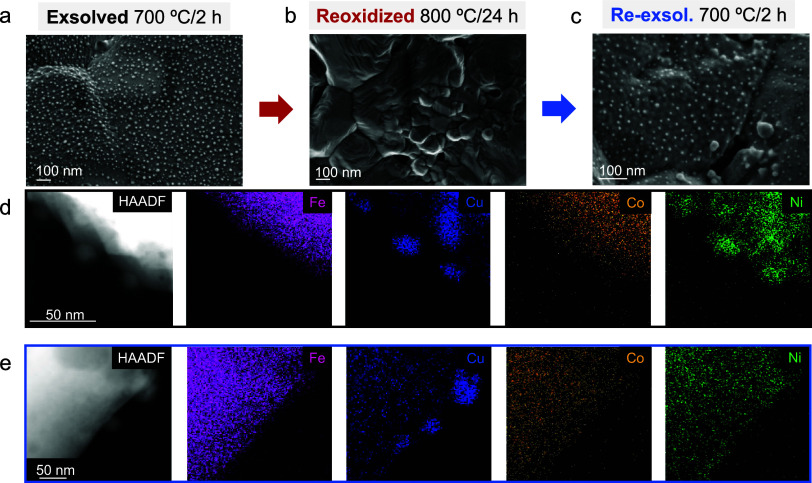
HRFESEM micrographs for (a) exsolution 700 °C/2 h, (b) oxidation of (a) at 800 °C/24 h, and (c) re-exsolution of (b) at 700 °C/2 h. HAADF-STEM and XEDS mapping analyses for exsolvable metals for (d) exsolution 700 °C/2 h and (e) re-exsolution 700 °C/2 h.

Given the treatment times employed, which might not reach an exsolution equilibrium state, longer exsolution treatments (24 h) were also considered for reversibility tests. Two sequential oxidation steps were performed in synthetic air, with the temperature set to 800 °C for 2 and 24 h. HRFESEM micrographs and HAADF-EDX for these processes are shown in Figure 6a–c and d–f, respectively. Exsolution for 24 h generates Janus-type nanoparticles that change upon oxidation. After 2 h in air, the particles become cubic-polyhedral, as seen in other studies,^45,55^ with Cu redissolving into the lattice, leaving spinel-like Fe, Ni, Co nanoparticles. Notably, Cu first regresses to bulk perovskite from these Janus nanoparticles. This is counterintuitive, since the oxidation thermodynamics (see Ellingham plot in Figure S4b) indicate that Cu has the lowest tendency to be oxidized when exposed to air. This surprising behavior might be related to the fact that the Cu forms one of the separated phases of the Janus-type nanoparticle. On the other hand, the formation of pyramidal NiO nanoparticles (see Figures S16 and S17) after 24-h oxidation is consistent with a lower tendency of Ni to oxidize compared to Co and Fe, and with previous reports from our group on reversible ternary alloyed NPs.^45^ This can be appreciated in Figure S16 which shows complete elemental mapping of all elements in the material. It is worth mentioning that, in this scenario, complete redissolution after 800 °C/24 h reoxidation was not achieved, contrarily to the previous case, which started from 700 °C/2 h exsolution, full reincorporation of the exsolved nanoparticles was appreciated after the same oxidation treatment.

**Figure 6:**
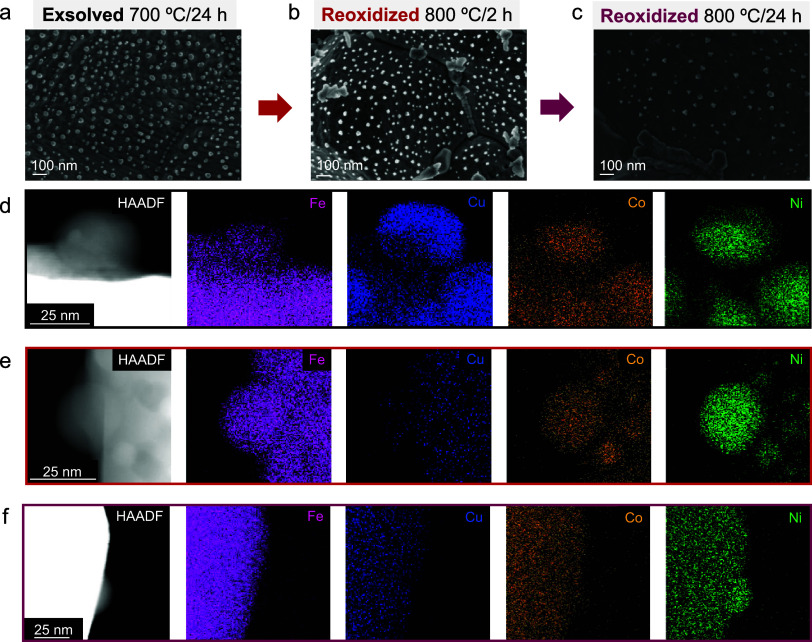
HRFESEM images and HAADF-STEM with XEDS mapping analysis for (a) and (d) exsolution 700 °C/24 h, (b) and (e) oxidation at 800 °C/2 h, and (c) and (f) oxidation at 800 °C/24 h.

Finally, Figure S18 shows the XRD results for the three states involved in these redox experiments. It can be observed that the 2 h treatment did not fully reoxidize the perovskite phase, as the lattice further contracted after 24 h in air. In both cases, the cubic phase is retained without detectable segregation of species.

Figure 7 depicts a schematic representation summarizing the presented findings. At 700 °C and exsolution times of 2 h, the NPs are mainly formed by Cu and Ni (Figure 2c), following the Ellingham reducibility trends (Figure S4). However, longer exsolution times (24 h) at the same temperature (700 °C) had remarkable implications over the nanoparticles’ composition, leading to the formation of Janus-type structures, composed by the **(i)** Cu-rich region and **(ii)** FeCoNi alloyed region. Furthermore, the initial exsolution treatment conditions not only greatly affected the nanoparticle composition but also has profound implications on the reoxidation process, thus on the reversibility of exsolution. Whereas mild exsolution conditions (700 °C, 2 h), led to full redissolution after 800 °C/24 h treatments and increased exsolution extents after re-exsolution, longer exsolution treatments (24 h) hindered nanoparticles’ reoxidation. In fact, after identical reoxidation treatment, complete redissolution was not achieved, with NiO particles remaining in the surface. Nanoparticle size could be ruled out as a cause since values remained stable. Only changes in the dispersion and composition were observed in the NPs with increasing times. The phase-separated Janus-type nanoparticles may therefore be responsible for hindering redissolution. Interestingly, and opposite to thermodynamic predictions, Cu species were the first to redissolve back to the host oxide from the exsolved nanoparticles. Then, and this time in accordance with the predictions, Fe and Co regressed back, leaving NiO pyramidal (see Figures S16 and S17) particles populating the perovskite surface. Based on the current data, it is not possible to determine the main cause of this behavior. A detailed analyses of the surface cation rearrangement might be needed to fully unveil the redissolution limitations.

**Figure 7:**
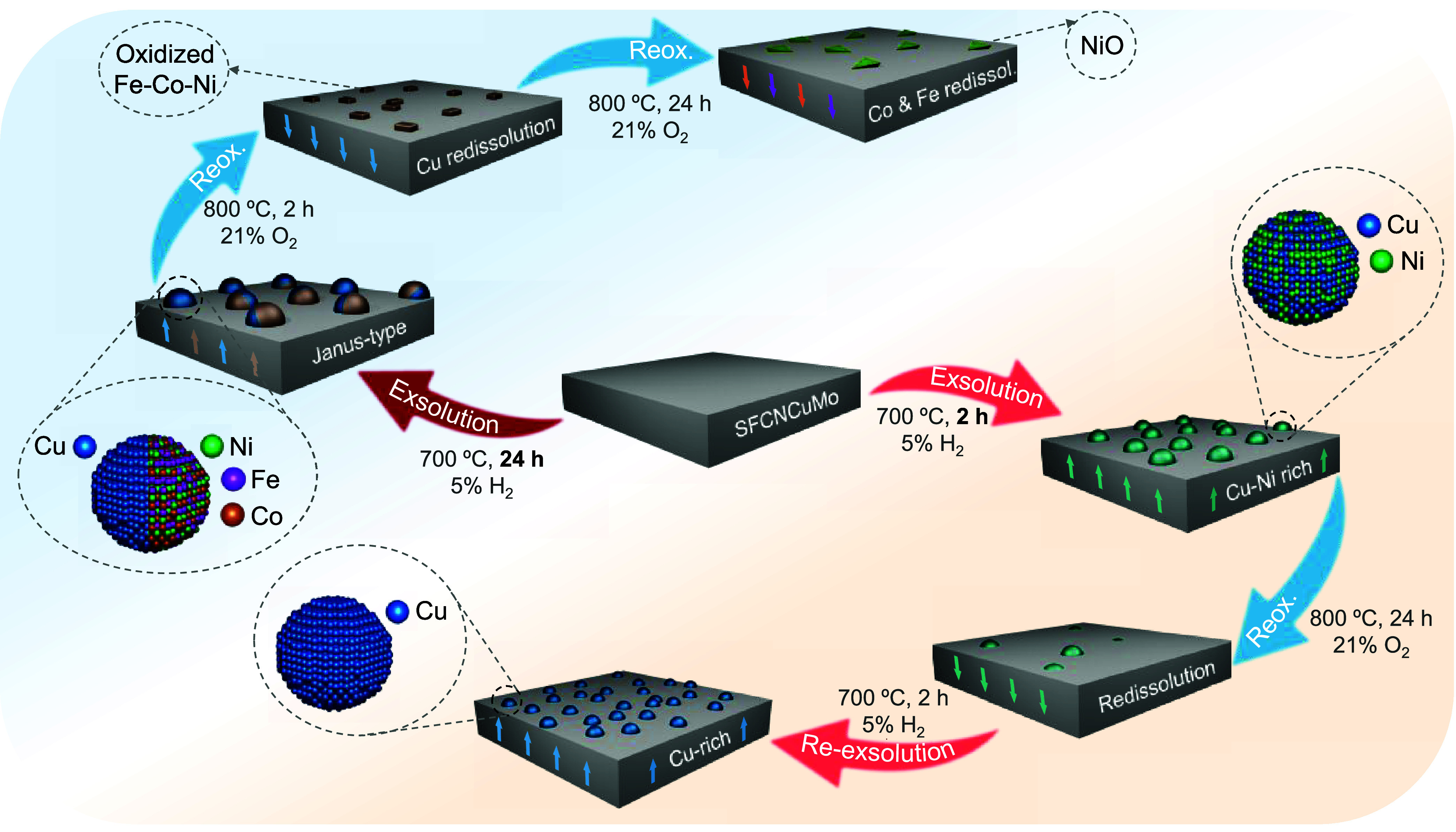
Schematic of Sr_2_Fe_1.2_Co_0.1_Ni_0.1_Cu_0.1_Mo_0.5_O_6−δ_ depicting the compositional and morphological changes occurring during exsolution–oxidation–re-exsolution processes.

### 2.5 Monitoring Exsolution Effects with Electrochemical Impedance Spectroscopy (EIS)

Next, the electrochemical performance of the SFCNCuMo complex oxide was tested in SOFC mode. For that purpose, asymmetric ScSZ electrolyte-supported cells were used as described in Figure S19a, composed of a state-of-the-art cathode (BSFCY) and an anode based on SFCNCuMo mixed with CGO (see Section 4). HRFESEM micrographs of the cell before the electrochemical characterization are shown in Figure S20, depicting the good adherence of the electrodes. EIS analysis was used to evaluate how exsolution affects the electrochemical performance at different temperatures, H_2_ %, and times. Figure S19b shows the *j–V* and power density curves at different temperatures (700–900 °C). The maximum power density obtained was 138 mW/cm^2^ at 900 °C. The modest performance observed in this test is attributed to **(i)** the thickness of the electrolyte (150 μm) and **(ii)** the resistance added by the required buffer layer (CGO) required to avoid cation interdiffusion between the electrode and the electrolyte.

EIS was conducted at 0, 2, 6, and 24 h of exsolution, with 0 h marking the initial hydrogen application to the electrode; consequently, SFCNCuMo has no nanoparticles at this stage. As the extent of exsolution increased over time, the polarization resistance was monitored over a period of 0 to 24 h. The spectra were fitted to an equivalent circuit *R*_*o*_(*R*_*HF*_ *Q*_*HF*_)­(*R*_*MF*_ *Q*_*MF*_)­(*R*_*LF*_ *Q*_*LF*_) formed by three contributions, *R*_*p*_ = *R*_*HF*_ + *R*_*MF*_ + *R*_*LF*_, where HF, MF, and LF refer to high, medium, and low frequencies, respectively. The fitting results shown in Figure 8a–8c and Table S2 indicate a decrease at HF (10^4^–10^2^ Hz) and MF (10^2^–10 Hz), while the LF contribution (<10 Hz) shows little variation. The scarce decrease at HF could be ascribed to charge-transfer processes, which are enhanced by the presence of metallic nanoparticles, thereby increasing the number of active sites for hydrogen oxidation.^56,57^ The main resistance decrease is primarily associated with mechanisms active at medium frequencies (MFs), in agreement with the progression of the exsolution process. As exsolution evolves, metal species are reduced, consuming electrons and generating oxygen vacancies at the electrode surface, thereby enhancing the surface activity. This is consistent with the work of Orera et al., who tested functionalized anodes with metal nanoparticles and observed a decrease in the polarization arc at medium frequencies due to improved diffusion and oxygen dissociation.^58^

**Figure 8:**
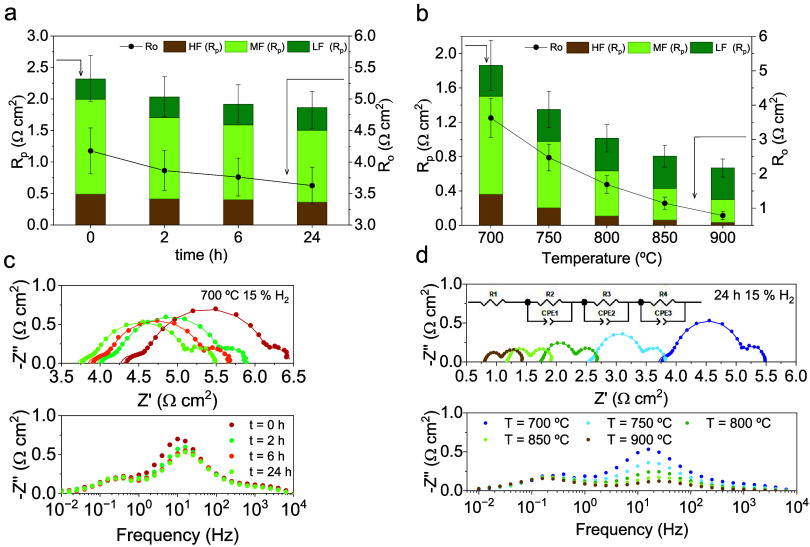
Total *R*_*o*_ and *R*_*p*_ and its contributions (*R*_*HF*_, *R*_*MF*_, *R*_*LF*_) in terms of (a) time of exsolution at 700 °C and (b) temperature of operation. Nyquist and Bode plots for EIS measurements in terms of (c) time of exsolution at 700 °C and (d) temperature of operation.

Figure 8b and d shows the ohmic (*R*_*o*_) and polarization (*R*_*p*_) resistances as a function of temperature from 700 to 900 °C. As expected, Arrhenius behavior is observed, *R*_*o*_ and *R*_*p*_ decreasing as temperature increases. The fitting results are shown in Figure 8d, which shows there is a decrease at high and medium frequencies, while the LF contribution does not vary significantly, as seen in the time tests. For this temperature study, the exsolution time of the anode was fixed at 24 h; thus, the results reflect thermally driven mechanisms activated by temperature. At medium frequencies, the observed process is primarily associated with elemental reactions occurring at the electrode surface, which are part of the surface-exchange kinetics. This MF contribution exhibits the greatest influence among the different frequency processes, with capacitance values on the order of 10^–2^ F·cm^– 2^ (Table S3). Such capacitance values suggest enhanced catalytic activity, consistent with an increased number of exsolved nanoparticles or reaction-active sites at the electrode surface. At high frequencies, the response is usually ascribed to electronic losses at the electrolyte/electrode or electrode/current collector interfaces, which become more pronounced with decreasing temperatures. Conversely, at low frequencies, the resistance contribution does not appear to be thermally activated, since it can be related to limiting diffusion phenomena within the electrode porous microstructure or gas phase. In addition, different percentages of hydrogen were tested on the anode side as depicted in Figure S21 and Table S4, which show the expected differences among the three result sets (15, 30, and 50% H_2_) from polarization resistance values decreasing from 1.47 to 0.81 Ω·cm^2^, respectively. To reduce fuel cell resistance, the atmosphere usually contains 50% H_2_. However, higher H_2_ levels could accelerate the exsolution process and hamper the time-resolved monitoring through EIS, so 15% H_2_ was selected for operation condition. To corroborate that effective exsolution is achieved under these electrochemical conditions, despite differences in setup, material configuration, and H_2_ concentration compared to powder studies, additional HRFESEM micrographs (700 °C, 2 h) are provided in Figure S22a. The cell performance is constrained by the electrolyte (*R*_*o*_) because it is electrolyte-supported, resulting in relatively high and stable ohmic resistance values over time, especially during exsolution studies. Consequently, additional research is necessary to enhance the cell overall performance. Finally, the evolution of the microstructure after the electrochemical test (all combined 200 h) is shown in Figure S22. The electrode microstructure is preserved after measurements, as shown by comparing both figures. HRFESEM micrograph of the fuel electrode after operation shows exsolution at the surface, although to a lower extent and with larger nanoparticles than in powder form. This confirms exsolution evolution during the tests in hydrogen, confirming its dynamic nature.^34^

## 3 Conclusions

This study successfully investigated the reversible exsolution of quaternary metallic nanoparticles from the Sr_2_Fe_1.2_Co_0.1_Ni_0.1_Cu_0.1_Mo_0.5_O_6‑δ_ complex perovskite oxide, highlighting a strong dependence on processing conditions. The results show that exsolution temperature dictates the primary composition of the nanoparticles: low temperatures favor Cu-rich particles, driven by the metal’s higher reducibility, whereas high temperatures yield Fe-rich particles. This dominance of Fe at elevated temperatures is a synergistic effect of enhanced reducibility and the higher cationic availability of Fe in the precursor material. The miscibility of the exsolved metals plays a crucial role in particle morphology, with limited solubility between Fe and Cu promoting the formation of phase-separated, Janus-type nanoparticles at intermediate compositions. Furthermore, the capacity for nanoparticle redissolution into the perovskite lattice is markedly dependent on the exsolution duration. Mild treatments enable a full recovery and subsequent re-exsolution, while prolonged treatments appear to stabilize the phase-separated morphology, which subsequently hinders the complete redissolution of the metals. Intriguingly, the redissolution sequence does not entirely follow thermodynamic predictions, as Cu species vanish first, before the predicted regression of Fe and Co leaving behind a stable NiO phase. The formation of Janus-type NPs altered the reversibility trends, leading to the formation of a wide variety of oxide-based shaped nanoparticles, indicating that redissolution is affected by phase separation. In summary, this work underscores the critical need to consider alloy thermodynamics and the kinetic effects of processing time to precisely control the composition and reversibility of multimetallic exsolved catalysts. These findings could guide in the design of advanced metal-supported electrocatalysts, functionalized via the exsolution method.

## 4 Experimental Methods

### 4.1 Materials Fabrication

Sr_2_Fe_1.2_Co_0.1_Ni_0.1_Cu_0.1_Mo_0.5_O_6‑δ_ (SFCNCuMo) was prepared by a modified Pechini method. The following metal precursors were used: Sr­(NO_3_)_2_ (99%, Merck), Fe­(NO_3_)_2_ ·9H_2_O (99.95%, Merck), Co­(NO_3_)_2_·6H_2_O (98%, Merck), Ni­(NO_3_)_2_·6H_2_O (98%, Fisher), Cu­(NO_3_)_2_·3H_2_O (98%, Merck), and (NH_4_)_6_Mo_7_O_24_·4H_2_O (99%, Merck). The proper amount of these precursors was mixed with citric acid (CA, 99%, Aldrich) in a molar ratio of metal:CA of 1:1.5, using 100 mL of distilled water and magnetically stirred at 60 °C until all the solids were dissolved. Subsequently, ethylene glycol (EG, 99%, Thermo-Fisher) was added to the solution in a CA/EG ratio of 2/3 wt % and the temperature was raised to 80 °C for water evaporation. Once enough water is evaporated, the solution is transferred to a drying oven at 220 °C overnight to form the gel, followed by its subsequent calcination. After this step, the powder is ground in an agate mortar and placed in a furnace at 1100 °C for 12 h, enough time and temperature to form the desired phase.

The exsolved materials were prepared by exposing the as synthesized (unless stated otherwise) materials to a 5% H_2_ flow in Ar at various temperatures and times. Oxidation tests for the exsolved materials are performed in the same furnace at 800 °C under a synthetic air flux. All heating ramps used during these procedures were 5 °C/min.

### 4.2 Physicochemical Characterization

Powder X-ray diffraction was performed on the as-synthesized materials as well as on the exsolved and reoxidized ones. These analyses were performed with a PANalytical CubiX diffractometer with Cu K_α1,2_ radiation and a Bragg–Brentano X’Celerator detector. Further analyses by Rietveld refinements were made with X’Pert HighScore Plus (version 3.0.0).

High-resolution field-emission scanning electron microscopy (HRFESEM) was employed to capture micrographs of the materials before and after exsolution treatments. To investigate possible morphological changes resulting from reduction treatments, a GeminiSEM 500 electron microscope from Zeiss Oxford Instruments was employed. These micrographs were analyzed with ImageJ software (version 1.52a) to obtain information about the size and population of the exsolved nanoparticles. These data were used to graph size histograms summarizing the information with Origin 2025b software. Energy-dispersive X-ray spectroscopy (XEDS) was used to obtain compositional information about the material and the exsolved nanoparticles. Scanning transmission electron microscopy (STEM) images were taken with a field-emission transmission electron microscope JEM 2100F from JEOL. This technique was also employed to corroborate the anchored nature of the exsolved nanoparticles.

Thermogravimetric measurements were performed using a Mettler Toledo TGS/SDT851e equipment, where, under a constant synthetic air flow, the temperature was increased from room temperature to 1000 °C under constant synthetic air flow.

### 4.3 Conductivity Measurements

Electrical conductivity is measured by the four-point DC technique using a Keithley 2601 programmable current source and a multichannel Keithley 3706 multimeter. For this aim, dense bars of the materials were sintered. Experimentally, powders of Sr_2_Fe_1.2_Co_0.1_Ni_0.1_Cu_0.1_Mo_0.5_O_6‑δ_ were mixed in an appropriate ratio with polyvinyl alcohol (99%, Sigma-Aldrich) in distilled water. This material was then pressed (30 kN) into rectangular bars for 3 min and then sintered at 1300 °C for 10 h. This temperature is chosen to achieve minimum porosity while avoiding melting of the bars. Silver ink was used to ensure adequate contact between the material and silver wires. Conductivity measurements were then performed under a 5% H_2_/Ar, air and N_2_ atmospheres from 850 °C to room temperature with a cooling rate of 1 °C/min. Therefore, the conductivity of the synthesized materials was evaluated in inert, oxidizing, and reducing conditions.

### 4.4 Cell Preparation

ScSZ ((Sc_2_O_3_)_*x*_(ZrO_2_)_1–*x*_, where *x* = 0.04–0.15), commercial electrolytes (Fuel Cell materials, 0.15 mm) were employed where a buffer layer was applied by the sputtering technique with a Pfeiffer Classic 250 with CESAR 133 RF power generator (13.56 MHz, 300 W), which facilitates the application of a thin layer, which is calcined at 1200 °C during 10 h giving a 100 nm layer. This is necessary due to the potential for cationic interdiffusion between the electrode and the electrolyte at sintering or operating temperatures. Electrodes were screen-printed on both sides with a 9 mm diameter. SFCNCuMo ink was prepared in a 60:40 proportion with Ce_0.8_Gd_0.2_O_2‑δ_ (CGO, Cerpotech), which will increase the ion conductivity while reducing the thermal expansion coefficient between the electrode and the buffer layer. This layer is sintered at 1000 °C for 2 h. On the other side, a composite of BSFCY (VICAR (Ba_0.5_Sr_0.5_)­(Co_0.8_Fe_0.2_)_0.9_Y_0.1_O_3‑δ_) and CGO in a ratio 40:60 was used with a sintering temperature of 925 °C for 2 h. Both electrode layers are approximately 30 μm thick. Finally, gold is applied as current collector on both sides and calcined at 875 °C for 1 h.

### 4.5 Impedance Spectroscopy

Due to setup restrictions, the minimum amount of H_2_ that could be used in the anode chamber of the fuel cell was 15%. This was not viewed as a drawback as it has been demonstrated that exsolution in cells often yields lower exsolution extents than in powder form. Therefore, a small increase in H_2_ percentage could compensate for this difference. 15, 30, and 50% H_2_ atmospheres in the anode were tested before electrochemical cell characterization.

The electrochemical measurements were performed with an open-flange test set up from Fiaxell SOFCs where the cell was placed inside the setup in a modified version of the proposed setup. Sealing was performed using a mica gasket, where a circular opening of intermediate size between the electrode and the electrolyte to isolate both chambers. The furnace is heated up to 700 °C, the same temperature at which the exsolution time-dependent tests are done, at a rate of 5 °C/min. Once the temperature is reached, the hydrogen flux is set to a 15% H_2_/N_2_ in the fuel electrode side and synthetic air in the air electrode side. The electrochemical tests were conducted as follows: at each specified time (0, 2, 6, and 24 h), the hydrogen flux was passed through a water trap, maintaining the water content to 3%. After 10 min of stabilization, electrochemical impedance spectroscopy (EIS) was measured with a VersaSTAT4 potentiostat. Once the impedance measurement was completed, the flux was again placed in a dry state, as the exsolution is studied and most well reported in dry media.^7,8,18^

### 4.6 Neutron Powder Diffraction

Neutron powder diffraction (NPD) patterns were collected on the D1B high-flux powder diffractometer at the Institut Laue–Langevin (ILL), Grenoble, France (CRG-2991).^59^ Data were recorded using neutrons with a wavelength of 1.28 Å over an angular range of 128° in 2θ. The powder sample was loaded into a vanadium holder with a diameter of 6 mm. To ensure reducing conditions, the experiment was conducted in a furnace under dynamic vacuum at approximately 6 × 10^–4^ mbar. The NPD patterns were analyzed by the Rietveld refinement method using the FullProf program.^60^

### 4.7 *In Situ* Near-Ambient Pressure X-ray Photoelectron Spectroscopy

Near-ambient pressure X-ray photoelectron spectroscopy (NAP- XPS) was performed at the BL24 CIRCE beamline located at the ALBA synchrotron using an electron energy analyzer Phoibos NAP 150 (SPECS). This beamline provides an energy resolution of *E*/Δ*E* ∼ 8000. Small deviations in photon energy were calibrated by referencing the Pt 4f_7/2_ peak of a grounded Pt foil to a binding energy of 71.1 eV.^45,49^ A pass energy of 20 eV and an energy step size of 0.1 eV were used, and these parameters were held constant for all spectra. It should be noted that the spectra were collected at kinetic energy of 400 eV, which corresponds to a photoelectron inelastic mean free path (IMFP) of 1.0 nm.^49^ For these *in situ* experiments, button cells were fabricated in which the working electrode was composed of the targeted perovskite oxide Sr_2_Fe_1.2_Co_0.1_Ni_0.1_Cu_0.1_Mo_0.5_O_6‑δ_ mixed with gadolinium doped-ceria, as to replicate the fuel cells used in the EIS experiments. The configuration of the cell is depicted in Figure S5 of the Supporting Information (SI). The button cells were held in place by a 0.12 mm thick Pt foil on the sample holder, which was also used for energy calibration. An IR laser (up to 150 W power) was used for sample heating. The *in situ* NAP-XPS measurements were performed in several stages. First, a cleaning step was performed by heating the sample up to 600 °C in a pure O_2_ atmosphere (0.2 mbar) to remove carbonaceous species from the surface. At this point, NAP-XPS was performed to record data in the pristine state. Subsequently, the temperature was lowered to 300 °C, from where the sample was slowly heated up to ca. 500 °C in a pure H_2_ atmosphere (0.1 mbar). The formation of metallic species was monitored by following changes in the valence band (VB). Once the VB showed changes indicative of a metallic phase (i.e., appearance of spectral intensity at the Fermi edge at 0 eV binding energy, BE), NAP-XPS was performed to record data in the reduced state (exsolution). A third step was performed by reoxidizing the cell again in O_2_ atmosphere (0.2 mbar) following the changes in Fe 2p, Ni 2p, Cu 2p, and Co 2p as to monitor exsolution reversibility *in situ*. A third step was performed by reoxidizing the cell in an O_2_ atmosphere (0.2 mbar) while monitoring the changes in Fe 2p, Ni 2p, Cu 2p, and Co 2p to study exsolution reversibility *in situ*. Photon energies were adjusted to maintain a kinetic energy of ∼400 eV. For this step, the temperature was kept constant to ensure that all core levels were measured under the same thermal conditions. Mo 3d, O 1s, and Sr 3d spectra were fitted with CasaXPS, using a Shirley background and a Gaussian–Lorentzian (30) line shape.

### 4.8 Thermodynamic Calculations

Thermodynamic simulations were performed using the software package HSC Chemistry 6.1 from Outotec Research Oy.

## Supplementary Material



## Notes

XRD and SEM data for temperature-dependent exsolution; nanoparticle size distribution histograms and influence of temperature on NP population; XRD and SEM images after exsolution at 6 h for different temperatures; Ellingham plots; XRD, SEM, and nanoparticle distribution histograms at different exsolution times; schematics and photographs of the cells used in *in situ* NAP-XPS; NAP-XPS spectra of C 1s (reduced and pristine); deconvoluted spectra (reduced and pristine) of Mo 3d, O 1s, and Sr 3d; NAP-XPS of Cu 2p_3/2_, Ni 2p_3/2_, Co 2p_3/2_, and Fe 2p_3/2_ reduced and reoxidized; XRD, EDX, and SEM after reoxidation. Histograms for NP size distribution after re-exsolution; XRD after exsolution, oxidation, and re-exsolution at different conditions; HRTEM, HAADF-STEM, and XEDX after oxidation; schematics, SEM cross-sections of the electrochemical cell and *j*–V curves at different temperatures; Nyquist plot at different H_2_ concentrations; HRFESEM of exsolution in the cell electrode; and parameters of EIS fittings (PDF)
