# Supplementary material for: Impact of Phase-Separated Janus-Type Formation on the Reversibility of Multicomponent Exsolved Nanoparticles from Complex Perovskites
Source: ACS Nano. 2026 May 5;20(19):14155–69. doi: 10.1021/acsnano.6c01371 (PMC13192328; doi:10.1021/acsnano.6c01371)
Supplement: Supplementary file 1 [file nn6c01371_si_001.pdf]

*Supporting Information for*

# Impact of Phase-separated Janus-type Formation on the Reversibility of Multicomponent Exsolved Nanoparticles from Complex Perovskites

Blanca Delgado-Galicia[1], Andrés López-García[1], Catalina Elena Jiménez[2], Rosario Suarez-Anzorena[2], Marcus Bär[2,3,4], Virginia Pérez-Dieste[5], Ainara Aguadero[6], Jose A. Alonso[6], Ines Puente-Orench,[7,8] Laura Almar[1], Alfonso J. Carrillo[1*], José Manuel Serra[1*]

[1]*Instituto de Tecnología Química (Universitat Politècnica de València-Consejo Superior de Investigaciones Científicas), 46022 València, Spain.*

[2]*Dept. Interface Design, Helmholtz-Zentrum Berlin für Materialien und Energie GmbH (HZB), Albert-Einstein-Str.15, 12489, Berlin, Germany*

[3]*Dept. X-ray Spectroscopy at Interfaces of Thin Films, Helmholtz-Institute Erlangen-Nürnberg for Renewable Energy (HI ERN), Albert-Einstein-Str. 15, 12489 Berlin, Germany*

[4]*Department of Chemistry and Pharmacy, Friedrich-Alexander-Universität Erlangen- Nürnberg (FAU), Egerlandstr. 3, 91058 Erlangen, Germany*

[5]*ALBA Synchrotron, Carrer de la Llum 2-26, 08290 Barcelona, Spain*

[6]*Instituto de Ciencia de Materiales de Madrid, CSIC, Cantoblanco, Madrid 28049, Spain;*

[7]*Diffraction Group, Institut Laue-Langevin, 71 Ave des Martyrs, CS 20156, 38042 Grenoble cedex 9, France*

[8]*Instituto de Nanociencia y Materiales de Aragón (INMA-CSIC), C/Pedro Cerbuna, 12, 50009 Zaragoza, Spain*

Corresponding authors: jmserra@itq.upv.es: alcardel@itq.upv.es

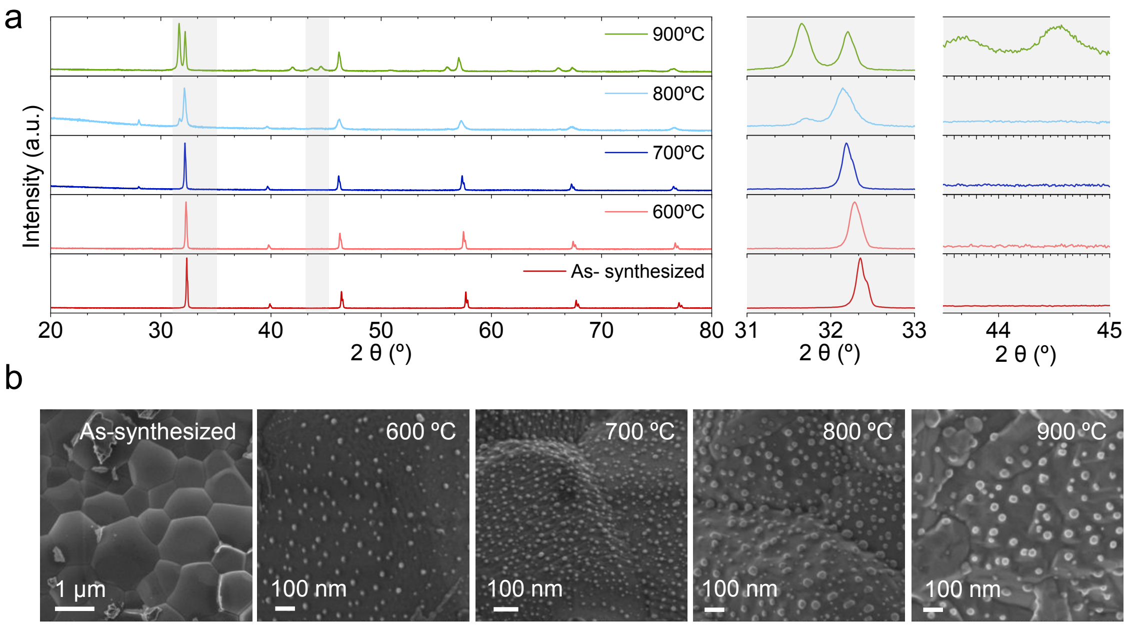


**Figure S1** Temperature dependent exsolution data for $Sr_2Fe_{1.2}Co_{0.1}Ni_{0.1}Cu_{0.1}Mo_{0.5}O_{6-\delta}$ pristine and exsolved materials at 600, 700, 800 and 900 ºC: (a) X-ray diffractograms. (b) HRFESEM micrographs.

**Table S1.** Temperature dependence phases data. Crystal phases, unit-cell volume, nanoparticle diameter, and population for $Sr_2Fe_{1.2}Co_{0.1}Ni_{0.1}Cu_{0.1}Mo_{0.5}O_{6-\delta}$ pristine and exsolved materials at 600, 700, 800, and 900 ºC.

| Sample label | Cubic phase (%) | Ruddlesden-Popper, RP (%) | Metallic alloy (%) | Unit-cell volume (Å$^3$) | NP. diameter (nm) | NP. population (Nanop./ μm$^2$) |
|---|---|---|---|---|---|---|
| **Pristine** | 100 | - | - | 478.8 | - | - |
| **Exs. 600 ºC** | 100 | - | - | 485.6 | 18.0 ± 5.9 | 148 ± 72 |
| **Exs. 700 ºC** | 100 | - | - | 487.3 | 15.6 ± 5.1 | 745 ± 140 |
| **Exs. 800 ºC** | 78.5 | 21.5 | - | 487.4 | 22.3 ± 9.4 | 410 ± 55 |
| **Exs. 900 ºC** | 5 | 83.6 | 11.4 | 487.0 | 36.5 ± 11.4 | 109 ± 11 |

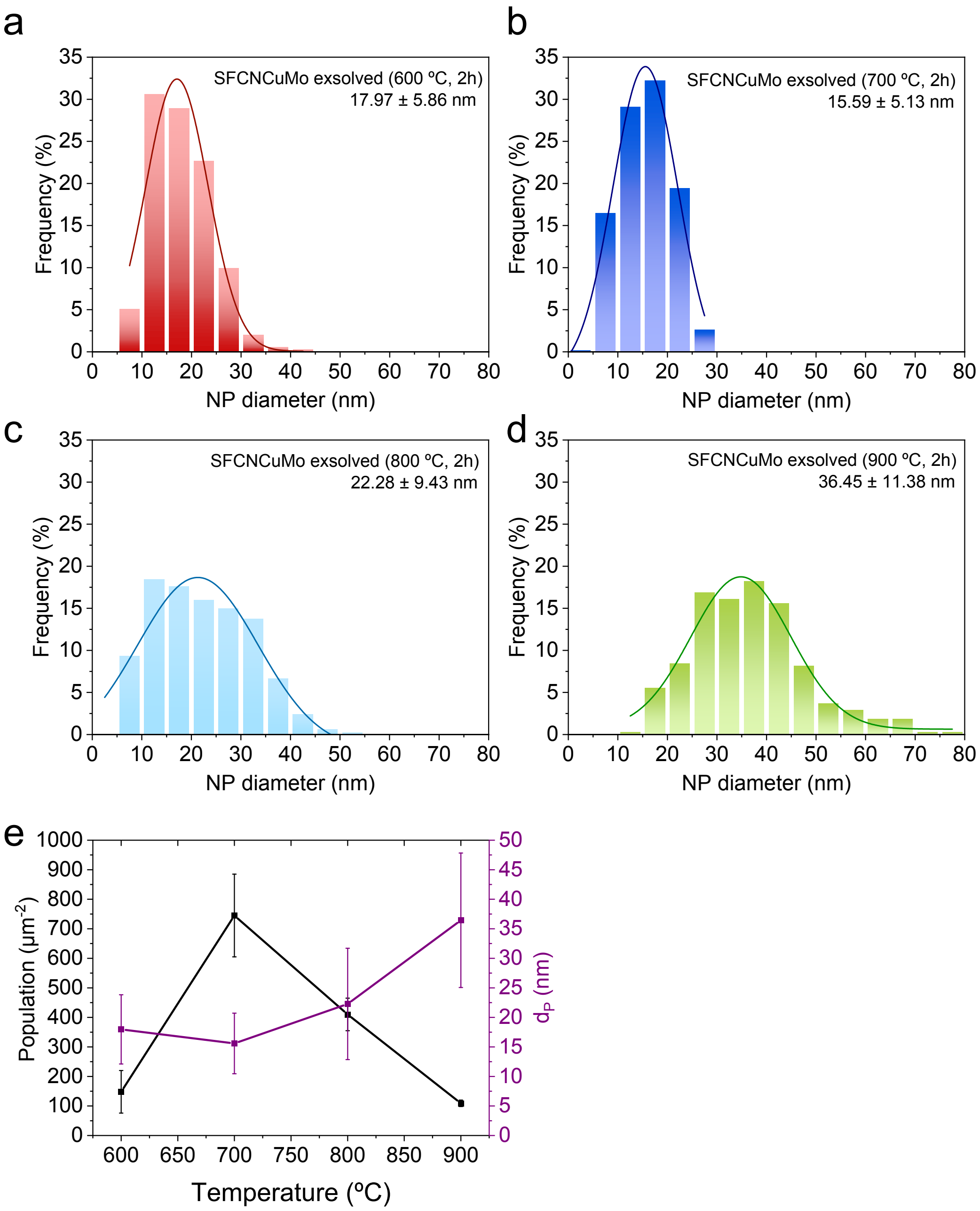


**Figure S2.** Histograms for nanoparticle diameter, mean size and standard deviation for samples exsolved at (a) 600, (b) 700, (c) 800, and (d) 900 ºC for 2 h. (e) Population and nanoparticle diameter variation.

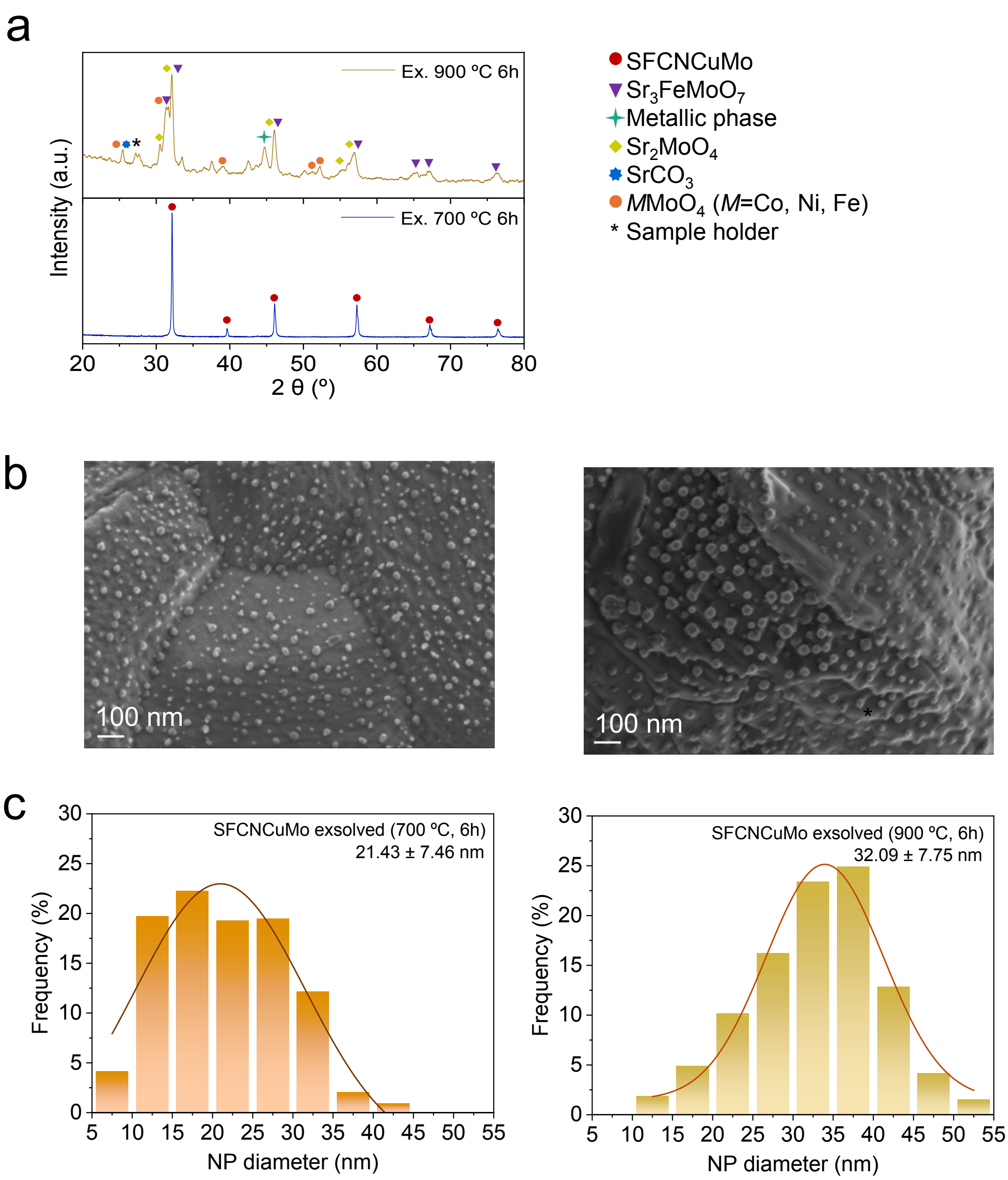


**Figure S3.** X-ray diffractograms (a), SEM images (b) and particle size distribution histograms (c) for SFCNCuMo exsolved for 6 hours at 700 ºC and 900 ºC.

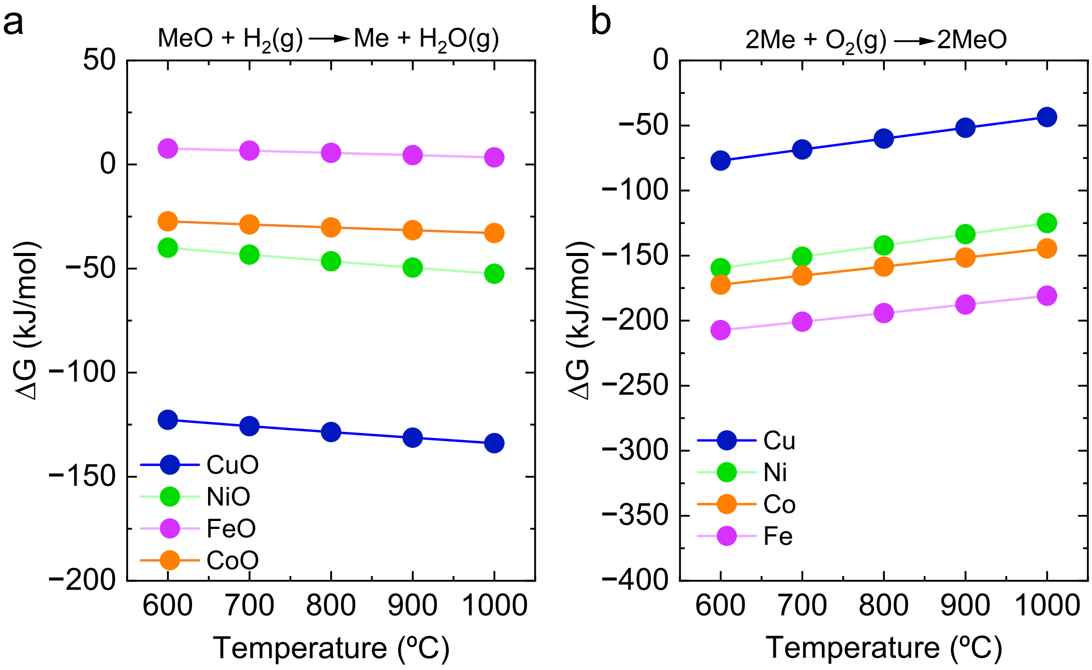


**Figure S4.** Ellingham diagram for the reduction of divalent transition metal oxides into their metallic elements (a) and the reverse oxidation reaction with oxygen (b). Data obtained with HSC software.

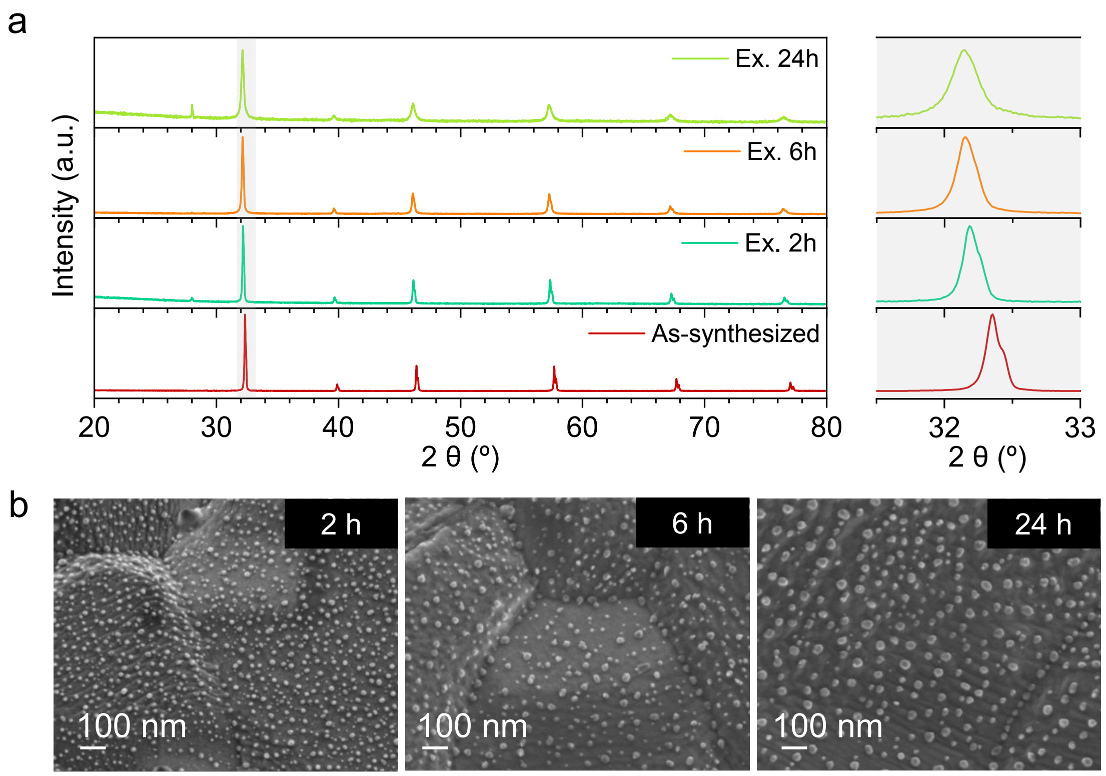


**Figure S5.** Time dependent exsolution data: (a) X-ray diffractograms, (b) HRFESEM micrographs for $Sr_2Fe_{1.2}Co_{0.1}Ni_{0.1}Cu_{0.1}Mo_{0.5}$ $O_{6-\delta}$ exsolved materials at 700 ºC for 2, 6 and 24 h.

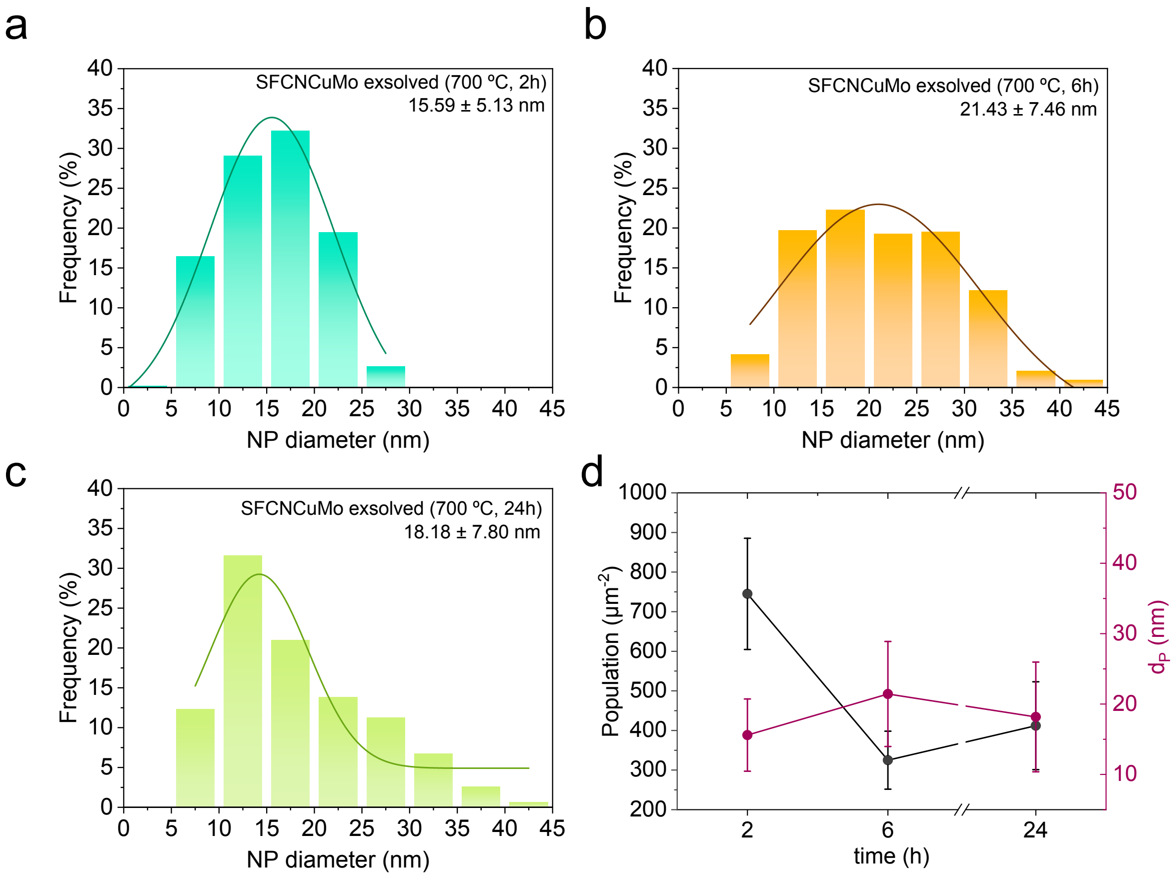


**Figure S6.** Histograms for nanoparticle diameter, mean size, and standard deviation for samples exsolved at 700 ºC for (a) 2, (b) 6, and (c) 24 h. (d) Population and nanoparticle diameter variation.

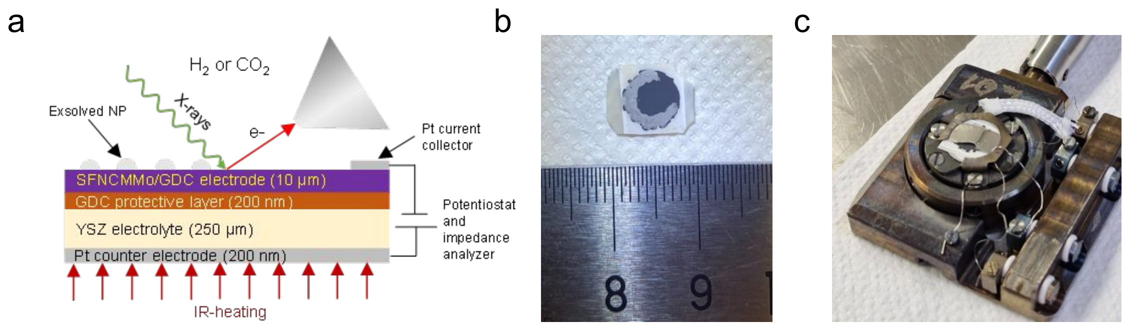


**Figure S7**: (a) Schematic of the electrochemical cell design used at CIRCE. (b) Top-view picture of one of the cells that was measured in the experimental campaign. (c) Picture of the device assembly where the electrochemical cell is mounted in the NAP-XPS sample holder with the pertinent electrical connections. A Pt foil was incorporated on top for calibration. The bottom electrical wire was covered by an alumina wool shield to prevent short-circuits. Additionally, between the bottom part of the cell and the sample holder, a thin zirconia-based ceramic lid was placed.

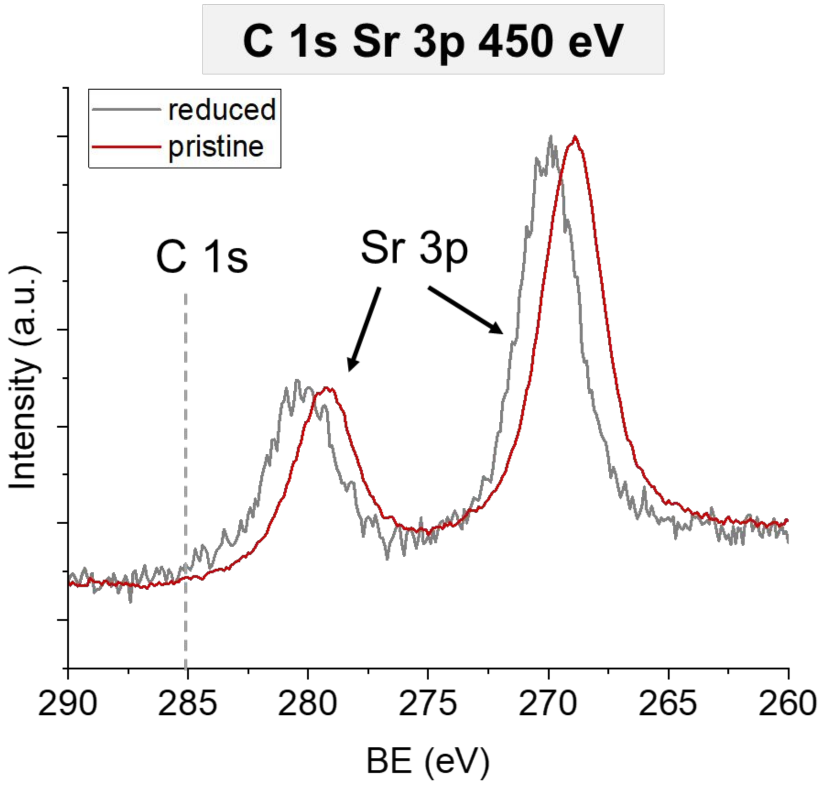


**Figure S8**. NAP-XPS spectra of C 1s-Sr 3p region at the pristine (0.2 mbar $O_2$), reduced (0.1 mbar $H_2$) states recorded using 450 eV photon energy. The dashed line indicates the C 1s BE of adventitious carbon, illustrating that the cleaning step in $O_2$ at 600 ºC helped in removing carbon species.

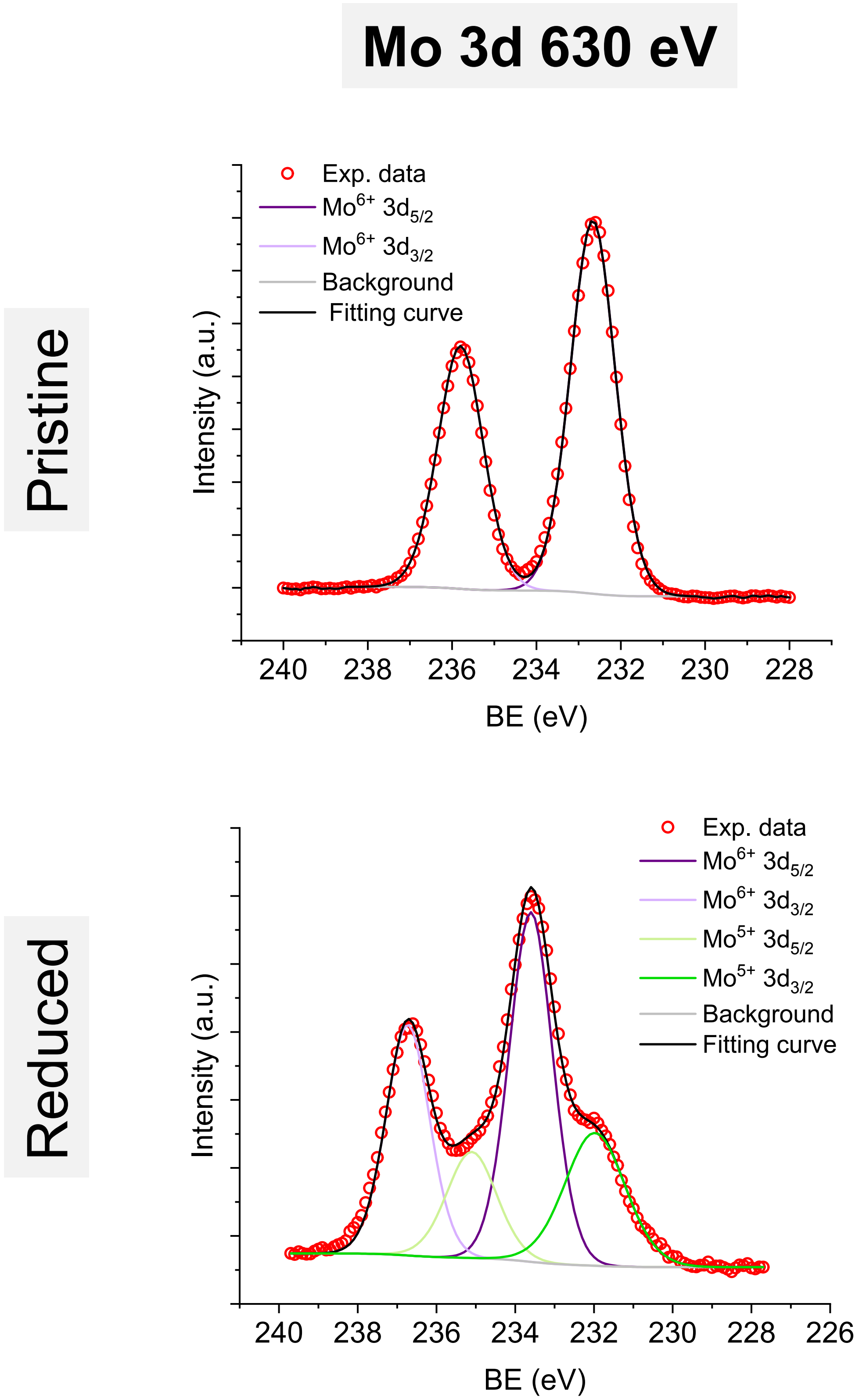


**Figure S9**. NAP-XPS spectra of Mo 3d at the pristine (0.2 mbar $O_2$), reduced (0.1 mbar $H_2$) states, recorded using 630 eV photon energy.

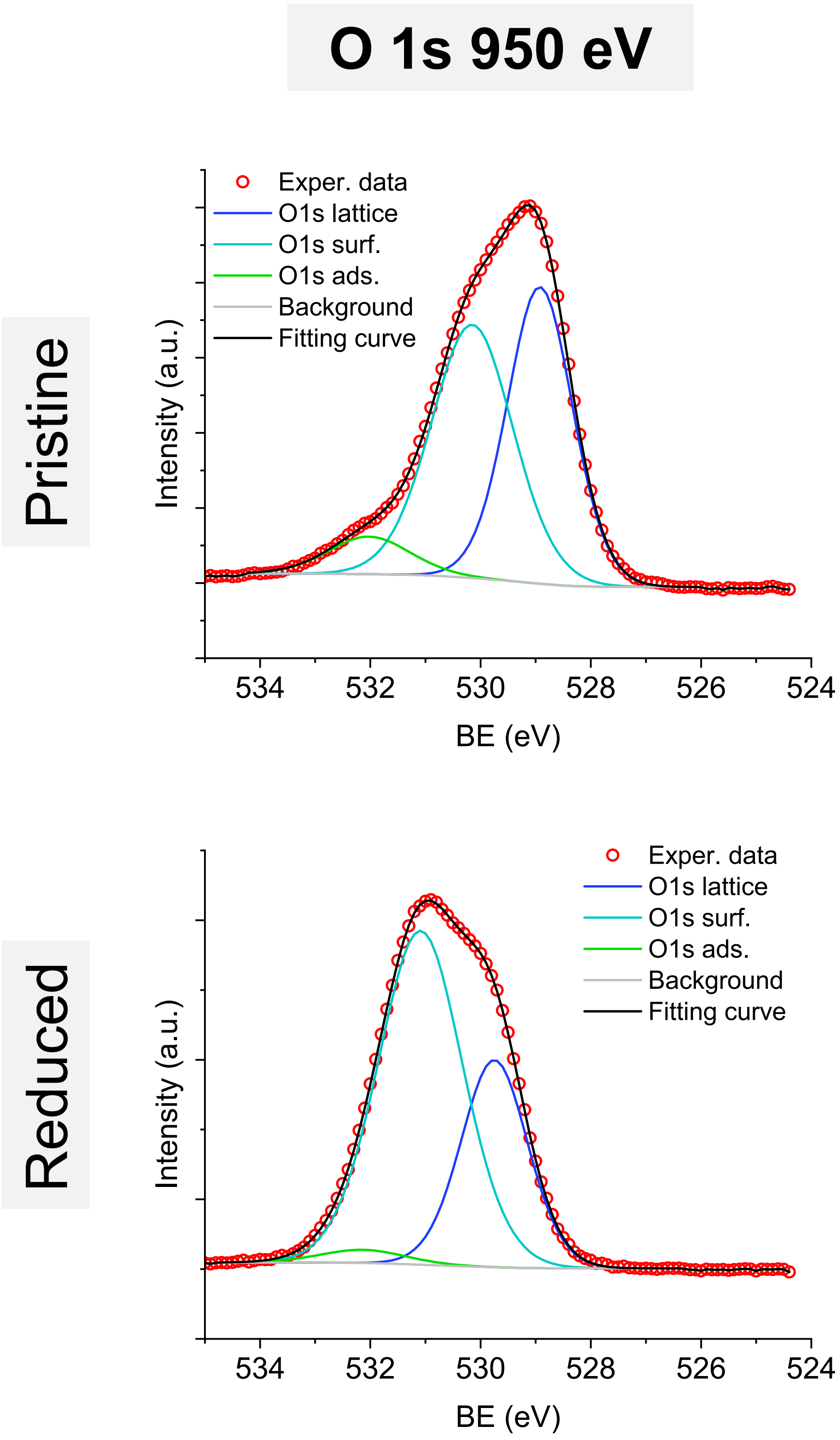


**Figure S10**. NAP-XPS spectra of O 1s at the pristine (0.2 mbar $O_2$), reduced (0.1 mbar $H_2$), recorded using 930 eV photon energy.

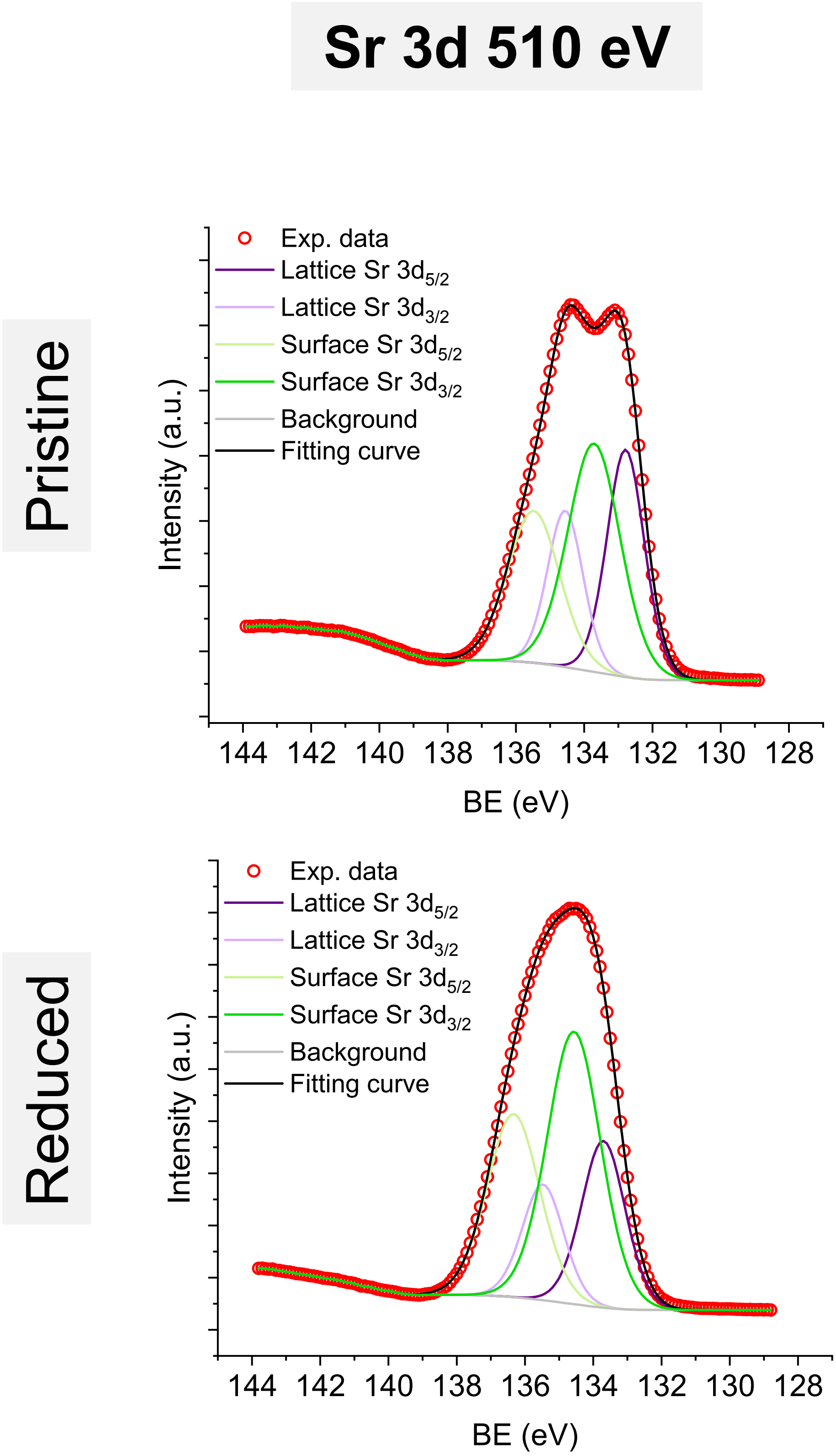


**Figure S11.** NAP-XPS spectra of Sr 3d at the pristine (0.2 mbar $O_2$), reduced (0.1 mbar $H_2$), recorded using 510 eV photon energy.

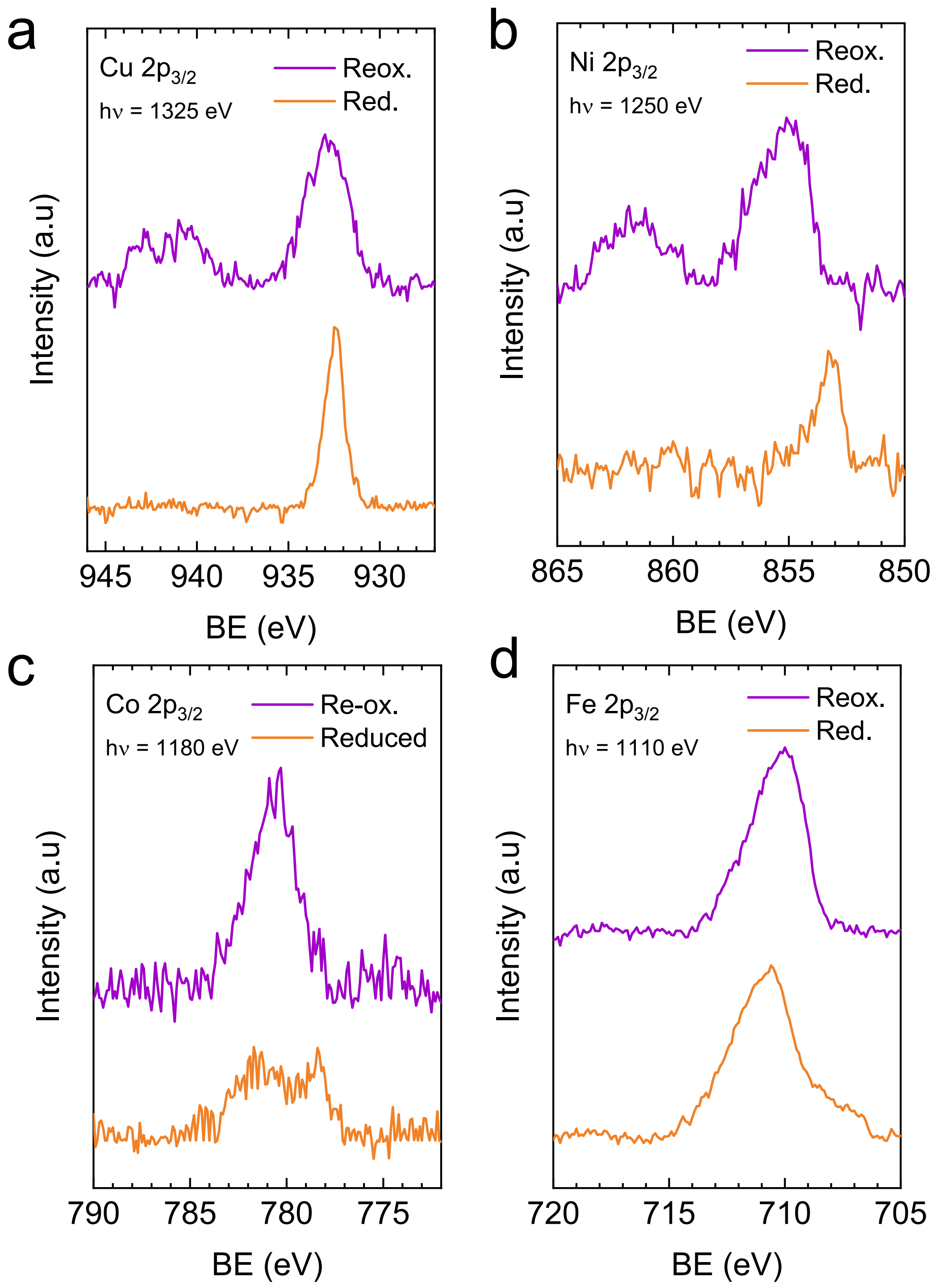


**Figure S12.** NAP-XPS spectra of (a) Cu $2p_{3/2}$, (b) Ni $2p_{3/2}$, (c) Co $2p_{3/2}$, and (d) Fe $2p_{3/2}$ at the, reduced (0.1 mbar $H_2$), and re-oxidized (0.2 mbar $O_2$) states.

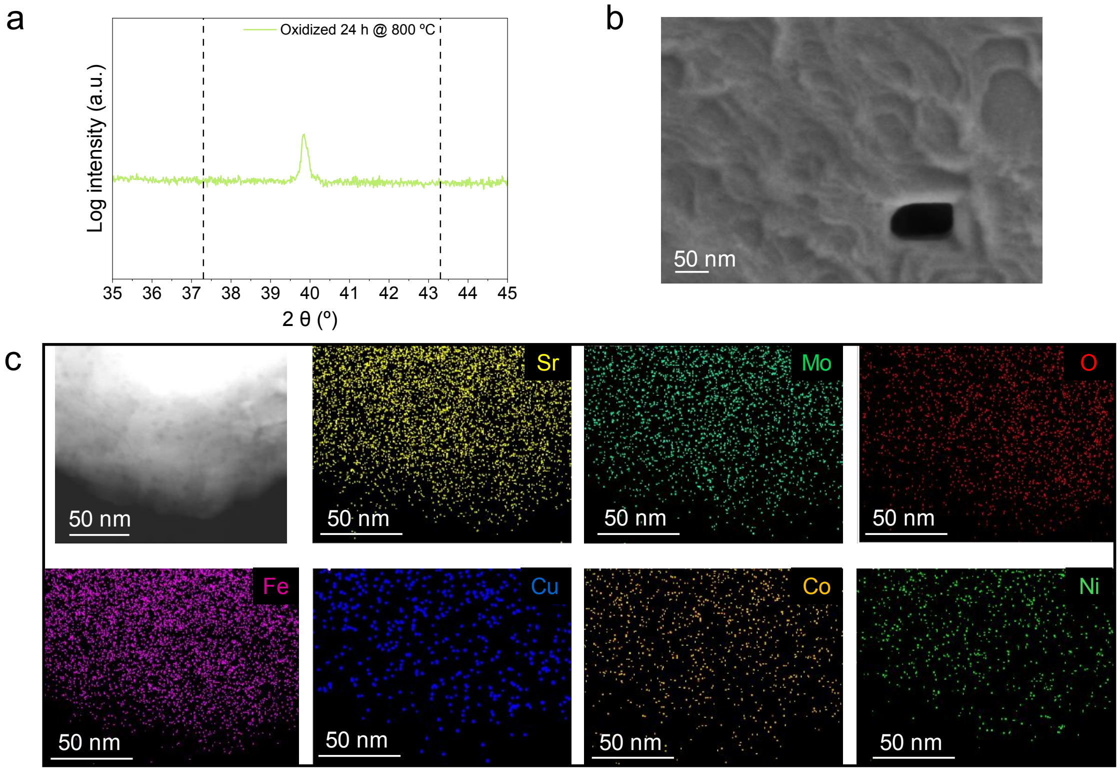


**Figure S13**. (a) XRD amplification of the re-oxidized material at 800 ºC / 24 h. Dashed lines correspond to NiO theoretical positions for (111) and (200) reflections (37.3 and 43.3 º, respectively). (b) High-magnification HRFESEM micrograph of re-oxidized material. (c) HAADF-EDX analyses showing the absence of NiO surface particles and the effective redissolution of exsolved species.

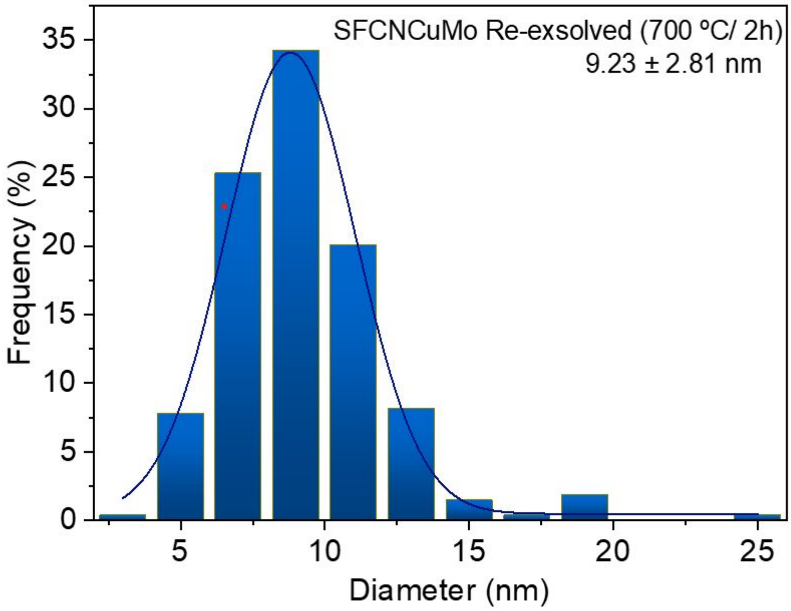


**Figure S14.** Histogram for re-exsolution at 700 ºC for 2 h after exsolution (700 ºC/2h) and reoxidation (800 ºC/24h).

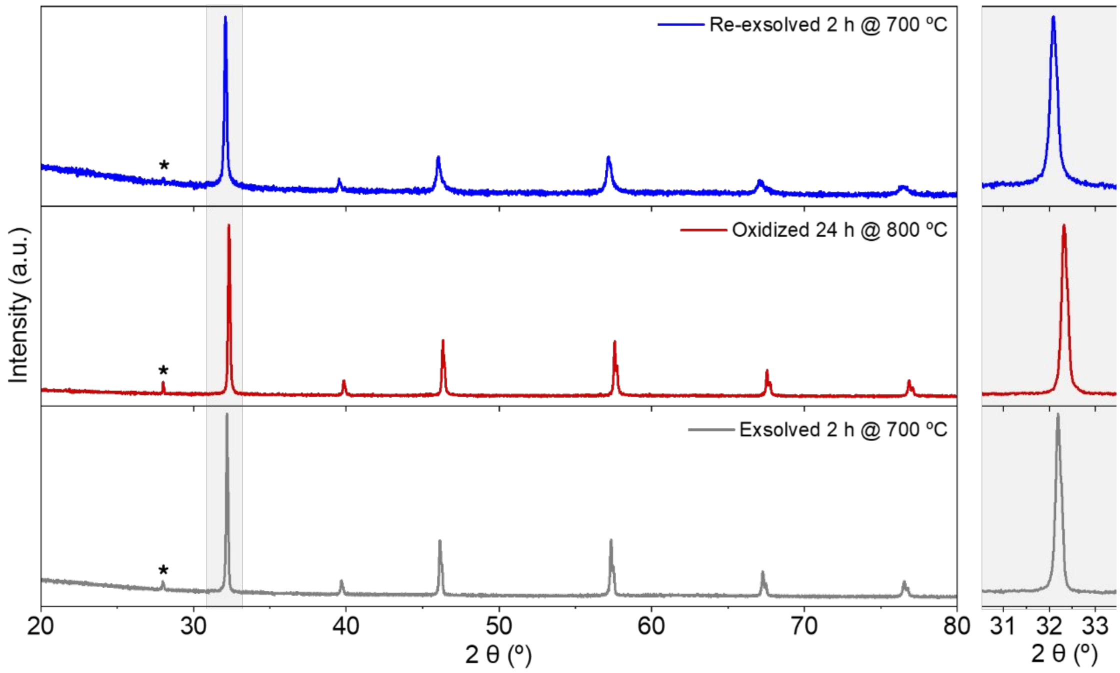


**Figure S15.** X-ray diffractograms from bottom to top of: SFCNCuMo exsolved at 700 ºC for 2 hours; this material oxidized at 800 ºC for 24 hours and finally re-exsolved at 700 ºC for 2 hours. Reflection at 28 º (*) corresponds to the sample holder.

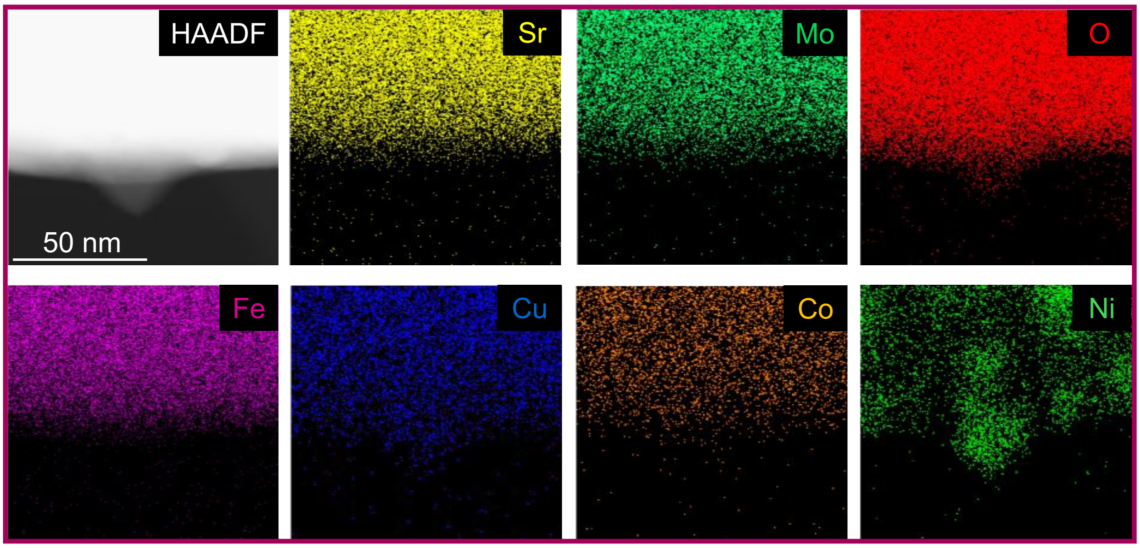


**Figure S16.** HAADF-STEM with XEDS mapping analyses of oxidation treatments at 800 ºC/24 h after exsolution at 700 ºC for 24 hours.

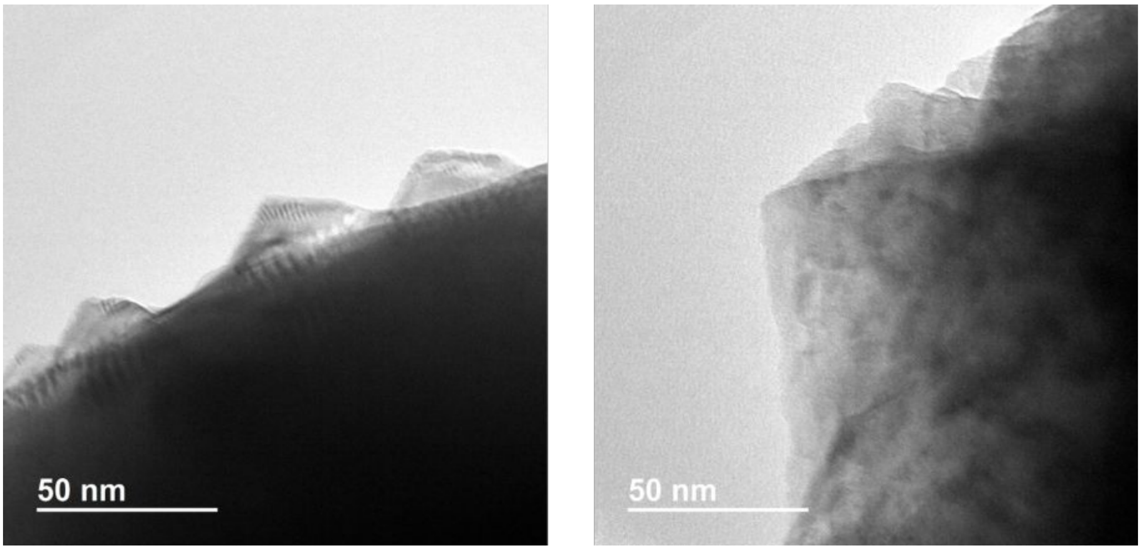


**Figure S17.** HRTEM micrographs of nanoparticles exsolved at 700 ºC for 24 h (left) and after oxidation at 800 ºC for 24 h (right).

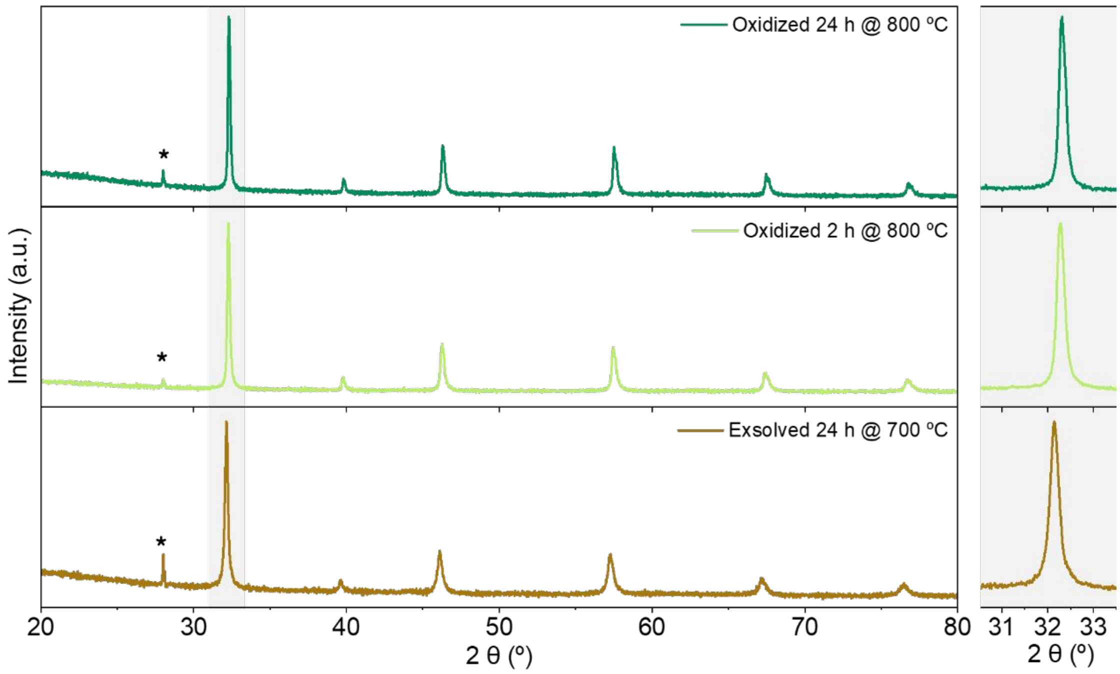


**Figure S18.** X-ray diffractograms from bottom to top of: SFCNCuMo exsolved at 700 ºC for 24 hours; this material oxidized at 800 ºC for 2 hours and 24 hours. Peak at 28 º (*) corresponds to the sample holder.

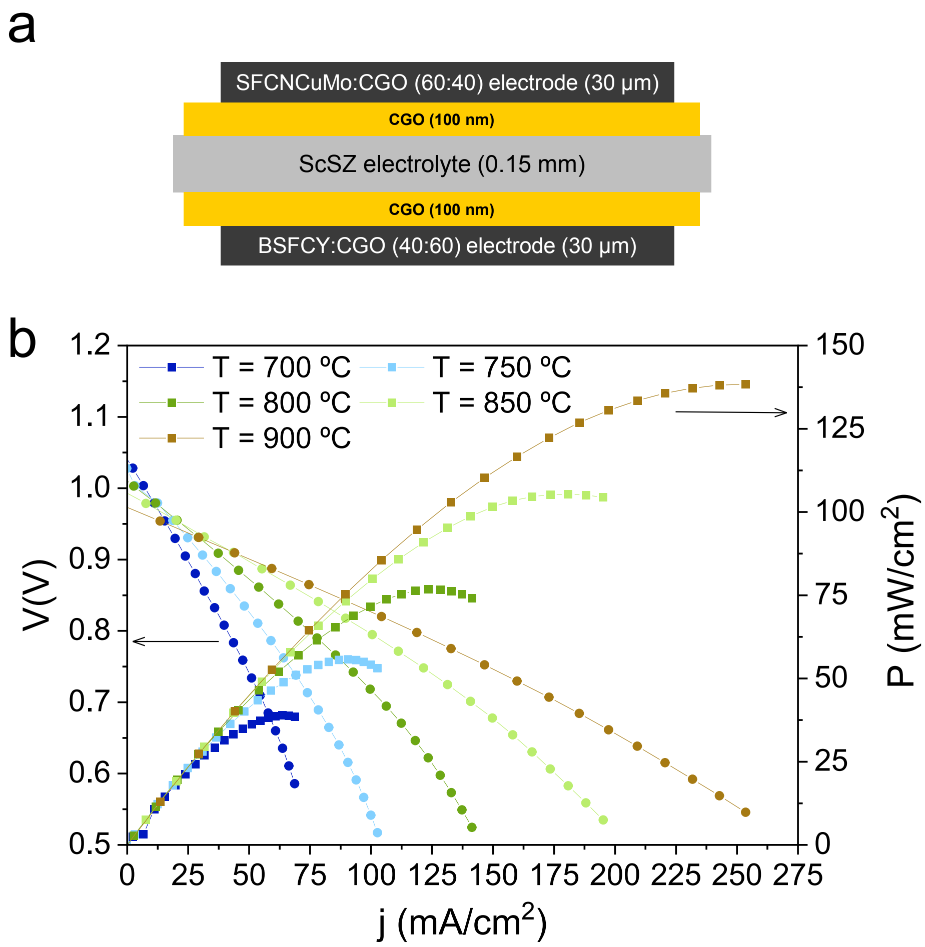


**Figure S19.** (a) Schematic of the SOFC electrochemically tested with exsolved SFCNCuMo anode. (b) *j*-*V* and power density curves at temperatures from 900 to 700 ºC.

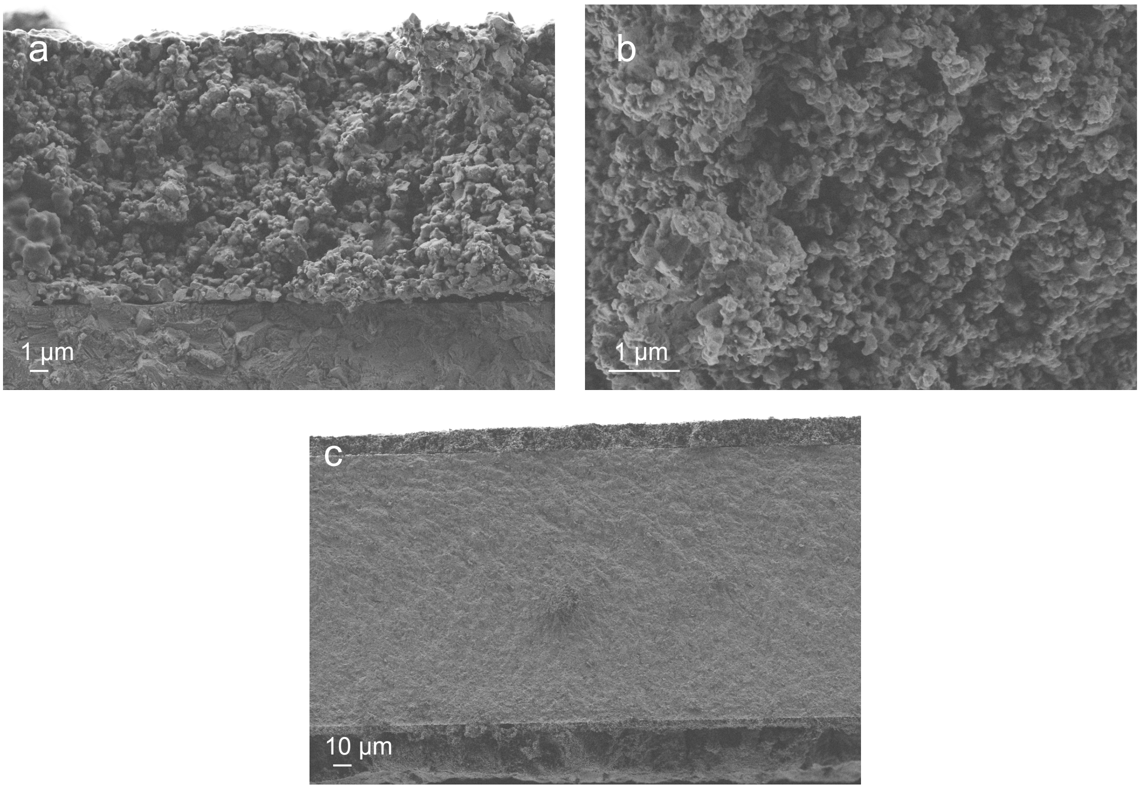


**Figure S20.** SEM images of the cell before electrochemical measurements of (a) SFCNCuMo:CGO fuel electrode. (b) BSFCY:CGO air electrode and (c) the entire cell, where the fuel electrode is on top and the air electrode at the bottom.

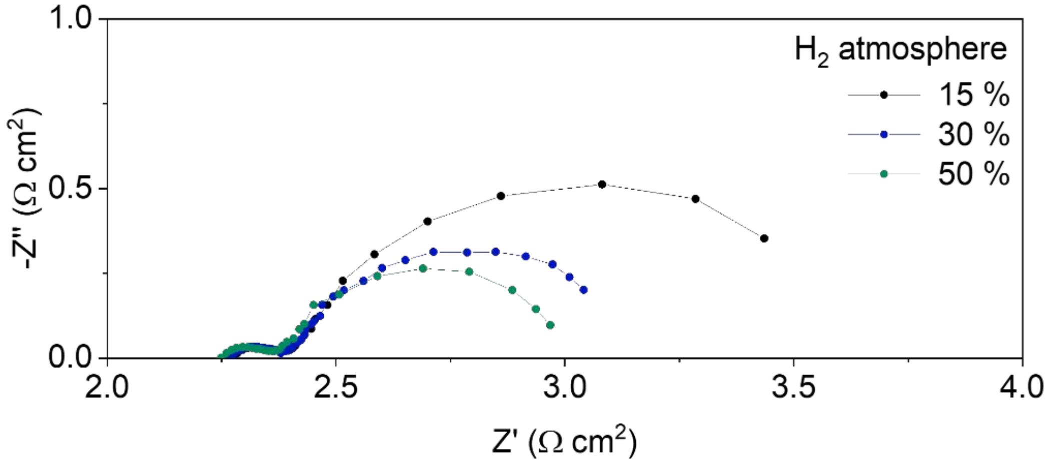


**Figure S21.** Nyquist plot of the SOFC tested with exsolved SFCNCuMo anode at 700 ºC for different $H_2$ atmospheres vs synthetic air.

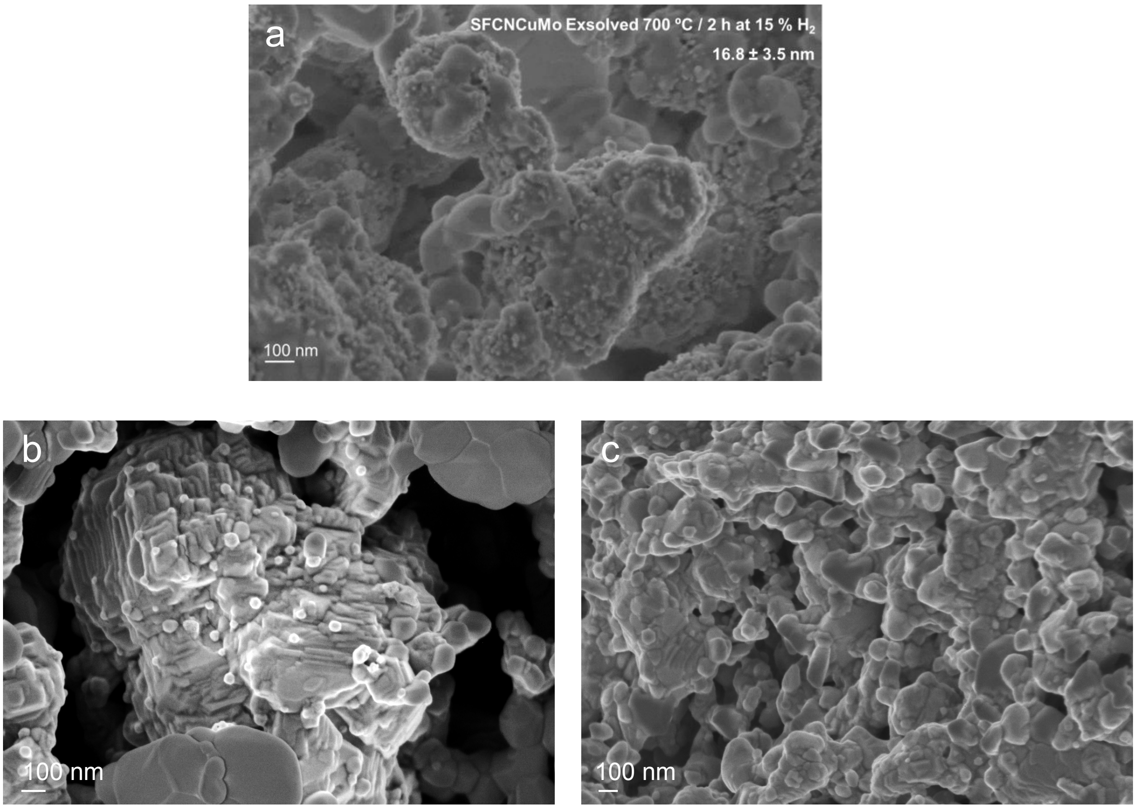


**Figure S22.** HRFESEM micrographs of (a) exsolved anode (SFCNCuMo:CGO) at 700 ºC, 2 h and 15%$H_2$/Ar, confirming the adequate exsolution in the membrane-assembled material, including nanoparticles' measured mean size, showing no significant differences with powder samples. HRFESEM micrographs of the cell electrodes after electrochemical measurements after 200 h of (b) SFCNCuMo:CGO fuel electrode where remaining exsolved nanoparticles can be appreciated -in lower extent than in powder test- and (c) BSFCY:CGO air electrode.

**Table S2.** Fit parameters of the equivalent circuit for the SOFC at different times of exsolution.

| t (h) | Ro ($\Omega$ cm$^{-2}$) | $R_{HF}$ ($\Omega$ cm$^{-2}$) | $Ceq_{HF}$ (F · cm$^{-2}$) | $R_{MF}$ ($\Omega$ cm$^{-2}$) | $Ceq_{MF}$ (F · cm$^{-2}$) | $R_{LF}$ ($\Omega$ cm$^{-2}$) | $Ceq_{LF}$ (F · cm$^{-2}$) |
|---|---|---|---|---|---|---|---|
| **0** | 4.17 | 0.56 | $6.11 \cdot 10^{-4}$ | 1.44 | 0.010 | 0.33 | 2.07 |
| **2** | 3.86 | 0.42 | $1.24 \cdot 10^{-4}$ | 1.29 | 0.012 | 0.33 | 2.01 |
| **6** | 3.76 | 0.40 | $1.27 \cdot 10^{-4}$ | 1.19 | 0.008 | 0.34 | 1.89 |
| **24** | 3.66 | 0.35 | $2.56 \cdot 10^{-4}$ | 1.12 | 0.009 | 0.36 | 1.75 |

**Table S3.** Fit parameters of the equivalent circuit for the cell at different temperatures.

| T (ºC) | $R_o$ ($\Omega$ cm$^{-2}$) | $R_{HF}$ ($\Omega$ cm$^{-2}$) | $Ceq_{HF}$ (F · cm$^{-2}$) | $R_{MF}$ ($\Omega$ cm$^{-2}$) | $Ceq_{MF}$ (F · cm$^{-2}$) | $R_{LF}$ ($\Omega$ cm$^{-2}$) | $Ceq_{LF}$ (F · cm$^{-2}$) |
|---|---|---|---|---|---|---|---|
| **700** | 3.66 | 0.35 | $2.56 \cdot 10^{-4}$ | 1.12 | 0.009 | 0.36 | 1.75 |
| **750** | 2.48 | 0.20 | $2.27 \cdot 10^{-4}$ | 0.77 | 0.011 | 0.37 | 1.92 |
| **800** | 1.68 | 0.11 | $2.85 \cdot 10^{-4}$ | 0.59 | 0.014 | 0.38 | 2.03 |
| **850** | 1.15 | 0.06 | $9.62 \cdot 10^{-4}$ | 0.36 | 0.018 | 0.38 | 2.19 |
| **900** | 0.78 | 0.04 | $2.06 \cdot 10^{-3}$ | 0.26 | 0.023 | 0.37 | 2.35 |

**Table S4**. Ohmic and polarization resistances of the SOFC of study for different $H_2$ atmospheres $H_2$ vs synthetic air.

| $H_2$ (%) | Ro ($\Omega$ · cm$^2$) | Rp ($\Omega$ · cm$^2$) |
|---|---|---|
| **15** | 2.23 | 1.47 |
| **30** | 2.22 | 0.97 |
| **50** | 2.21 | 0.81 |
